# The Radical Anion, Dianion and Electron Transport Properties of Tetraiodotetraazapentacene

**DOI:** 10.1002/chem.202201919

**Published:** 2022-10-25

**Authors:** Thomas Wiesner, Zhu Wu, Jie Han, Lei Ji, Alexandra Friedrich, Ivo Krummenacher, Michael Moos, Christoph Lambert, Holger Braunschweig, Benjamin Rudin, Hilmar Reiss, Olena Tverskoy, Frank Rominger, Andreas Dreuw, Todd B. Marder, Jan Freudenberg, Uwe H. F. Bunz

**Affiliations:** ^1^ Organisch-Chemisches Institut Ruprecht-Karls-Universität Heidelberg Im Neuenheimer Feld 270 69120 Heidelberg Germany; ^2^ Institut für Anorganische Chemie and Institute for Sustainable Chemistry & Catalysis with Boron Julius-Maximilians-Universität Würzburg Am Hubland 97074 Würzburg Germany; ^3^ Institut für Organische Chemie Julius-Maximilians-Universität Würzburg Am Hubland 97074 Würzburg Germany; ^4^ Interdisziplinares Zentrum für Wissenschaftliches Rechnen Physikalisch-Chemisches Institut Ruprecht-Karls-Universität Heidelberg Im Neuenheimer Feld 205 69120 Heidelberg Germany; ^5^ Present Adress: State Key Laboratory of Coordination Chemistry Jiangsu Key Laboratory of Advanced Organic Materials Chemistry and Biomedicine Innovation Center (ChemBIC) School of Chemistry and Chemical Engineering Nanjing University Nanjing 210023 P. R. China

**Keywords:** azaacenes, semiconductors, solid-state packing

## Abstract

Tetraiodotetraazapentacene **I_4_TAP**, the last missing derivative in the series of halogenated silylated tetraazapentacenes, was synthesized via condensation chemistry from a TIPS‐ethynylated diaminobenzothiadiazol in three steps. Single and double reduction furnished its air‐stable monoanion and relatively air‐stable dianion, both of which were characterized by crystallography. All three species are structurally and spectroscopically compared to non‐halogenated **TAP** and **Br_4_TAP**. **I_4_TAP** is an n‐channel material in thin‐film transistors with average electron mobilities exceeding 1 cm^2^ (Vs)^−1^.

## Graphical abstract

**Filling the gap** in the series of halogenated azaacenes. Tetraiodotetraazapentacene **I_4_TAP** is prepared and characterized. Its stable radical anion and high mobilities in organic field‐effect transistors make **I_4_TAP** a formidable n‐type semiconductor.

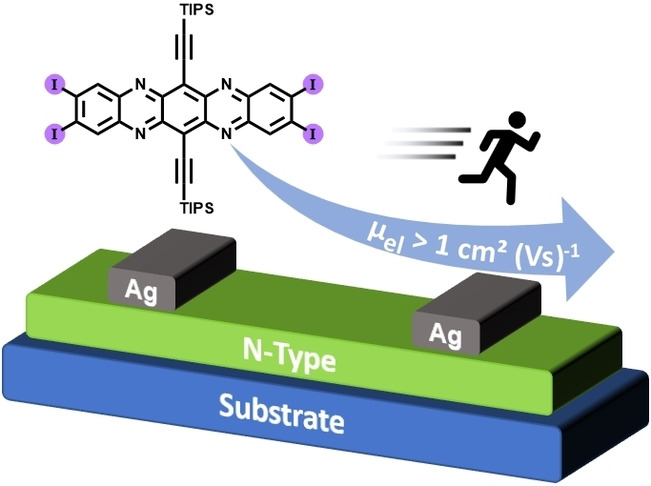

## Introduction

**Figure 1:**
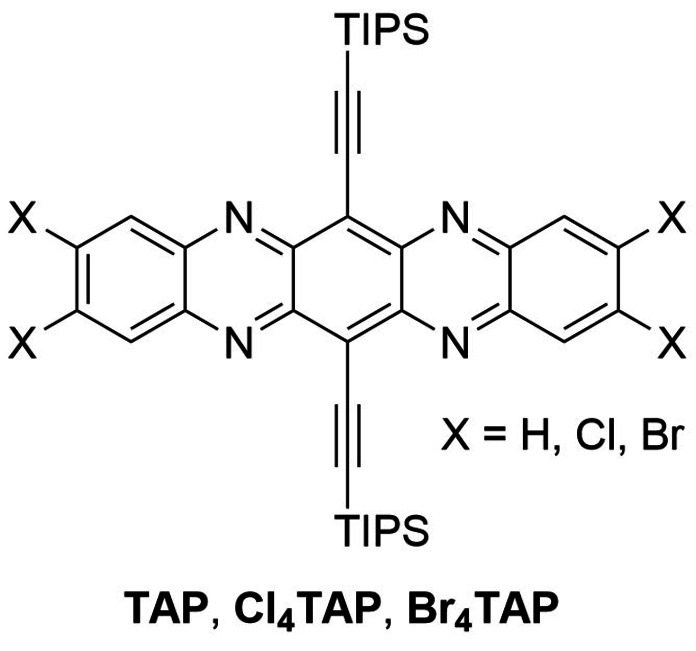
Previously published tetraazapentacene derivatives. For **TAP** and **Br_4_TAP**, radical anions were reported.[^10^, ^21^]

Herein we present tetraiodotetraazapentacene **I_4_TAP**, its radical anion and dianion, both of which are stable crystalline species. Some organic logic circuits^[1]^ require the combination of field‐effect transistors (FETs) based on robust p‐type^[2]^ and n‐type^[3]^ semiconductors – development of the latter still needs to catch up with their p‐type counterparts.^[4]^ Among small molecules, rylene diimides,^[5]^ tetraazaperopyrenes,^[6]^ and N‐heteroacenes are attractive electron transporters.^[7]^ Their most prominent representative, TIPS‐ethynylated tetraazapentacene **TAP**, was synthesized in 2009 (Figure 1, X=H).^[8]^ Later, halogenated derivatives **Cl_4_TAP** ^[9]^ and **Br_4_TAP** ^[10]^ were prepared which outperform **TAP** with increased electron mobilities[^10^, ^11^] in OFETs.^[12]^ The superior properties of the tetrahalides are related to improved charge distribution in the radical anions, the higher electron affinity of the neutral compounds, the increased transfer integrals,^[13]^ reduced reorganization energies,^[14]^ and, probably, to the increased persistence of the radical anions^[10]^ in air.^[15]^

## Results and Discussion

**Scheme 1:**
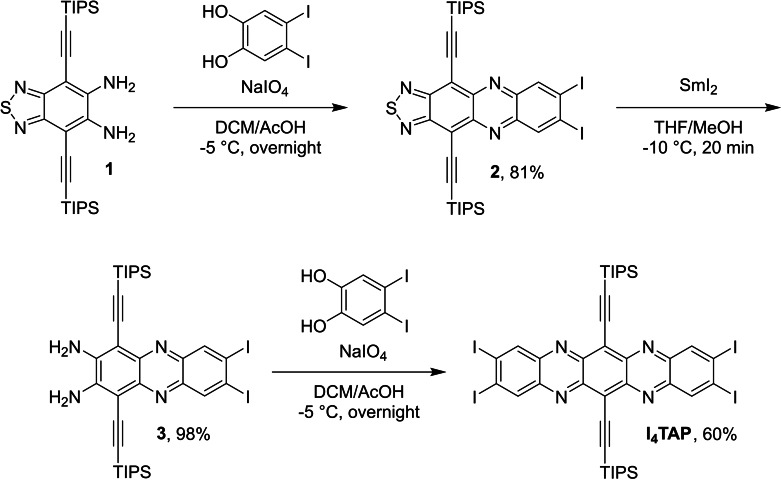
Synthesis of **I_4_TAP**.

**Scheme 2:**
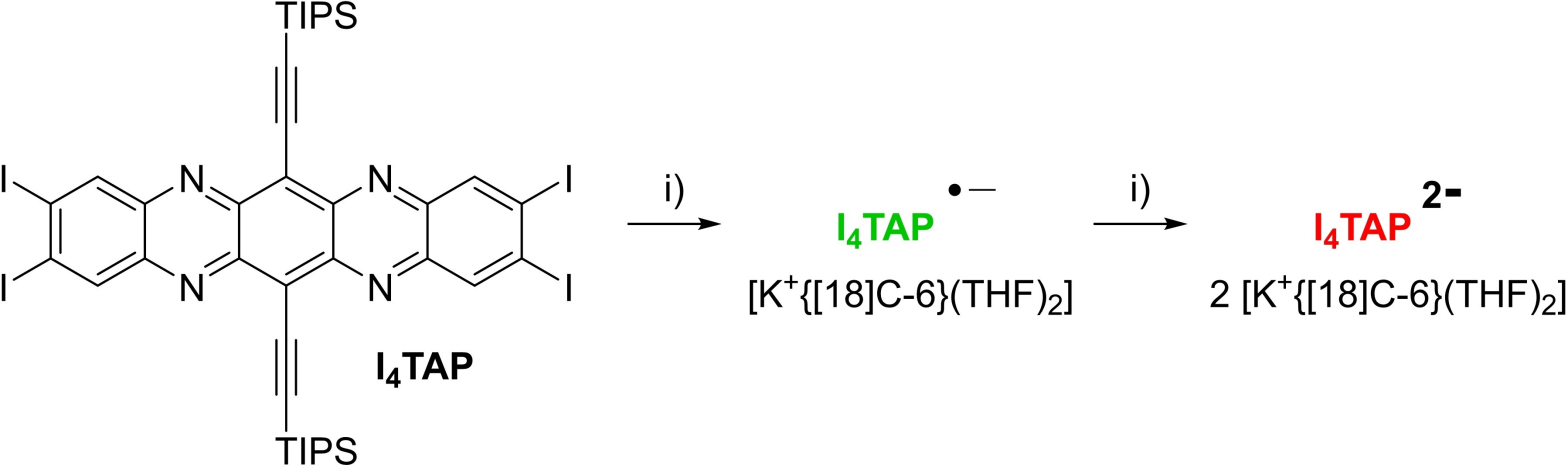
Synthesis of the radical anion and dianion of **I_4_TAP**. i) [K(18‐crown‐6)(THF)_2_] anthracenide in THF.

We describe the preparation and single electron reduction of the tetraiodo derivative **I_4_TAP** ^[16]^ (Scheme 1) starting from diamine **1** ^[17]^ and 4,5‐diiodocatechol, obtained from commercially available diiodoveratrole via BBr_3_‐induced ether cleavage.^[18]^ After oxidation of the veratrole with sodium periodate and condensation with **1**, the resulting thiadiazole **2** was deprotected by SmI_2_ (−10 °C) furnishing diamine **3** in near‐quantitative yield (98 %). The second condensation gave **I_4_TAP** as a crystalline material in 60 % yield. Key was performing both condensations at −5 °C due to the instability of the *ortho*‐quinone generated in situ, unlike previously used halogenated *ortho*‐quinones.[^9^, ^10^] Treating THF solutions of **I_4_TAP** with one equivalent of potassium anthracenide [18‐crown‐6] furnished **I_4_TAP^⋅−^** (Scheme 2), which was crystallized from THF and pentane to give specimens suitable for X‐ray diffraction analysis. **I_4_TAP** reacted with two equivalents of the anthracenide reagent to form **I_4_TAP^2−^** which was also crystallized from THF and pentane.

**Figure 2:**
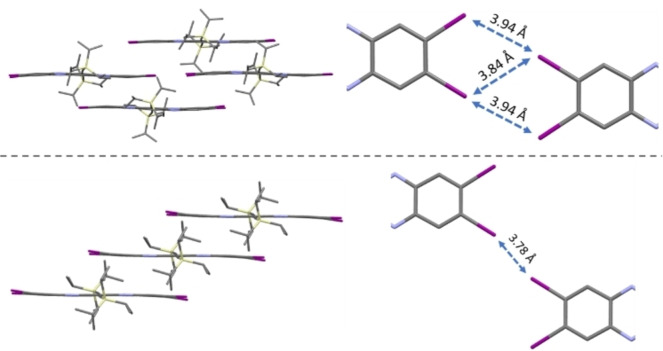
Different packing modes for **I_4_TAP**. Top: 2D brickwall packing (structure A, measured at 100 K) *P* $\bar{1}$ , *a*=10.6183(18) Å, *b*=18.084(3) Å, *c*=18.919(3) Å, *α*=110.634(13)°, *β*=94.523(14)°, *γ*=94.654(14)° with water molecules omitted for clarity. Bottom: 1D staircase (structure B, measured at 200 K) *P* $\bar{1}$ , *a*=7.6849(6) Å, *b*=9.8631(7) Å, *c*=14.5286(11) Å, *α*=86.0743(11)°, *β*=86.4298(12)°, *γ*=86.3349(11)°.

**I_4_TAP** crystallized in two different crystal structures (Figure 2): a brick‐wall (structure A; non‐dried chloroform, room temperature), and a staircase‐type packing (structure B; obtained after crystallization at different temperatures with different solvents combined with DCM or chloroform). Note that water co‐crystallized in structure A (see Supporting Information, Figure S18) as a result of crystallizing under ambient conditions. The structures exhibit two types of iodine‐iodine interactions that govern the packing.^[19]^ Within the brick‐wall there are three weaker iodine‐iodine short contacts on each side of the molecule, while within the staircase a single stronger interaction, 0.18 Å shorter than twice the van‐der‐Waals distance of I_2_, is present (Figure 2, bottom). Wavefunction analyses for the adjacent monomers revealed that the iodine−iodine interactions could be attributed to a σ‐hole interaction with substantial contributions from both electrostatic and dispersion attraction (Supporting Information, Section 6.5, Figure S25). In **Cl_4_TAP**, Cl−Cl distances do not suggest strong interactions (see Supporting Information, Figure S20). We could not reproduce the formation of structure A, even in wet chloroform; it might be a kinetic product. Surprisingly, when fabricating thin‐films on alkyl‐SAM‐coated surfaces under ambient conditions, only structure A was observed *via* grazing incidence diffraction (see Figures 2 and S21).

The bond lengths and bond angles of **I_4_TAP** are in excellent accord with expected values and do not differ significantly between the two packings (see Supporting Information, Figure S19). All alkyne groups deviate slightly from linearity with Si−C≡C and C−C≡C angles ranging from 175°–171° and 178°–174°.

**Figure 3:**
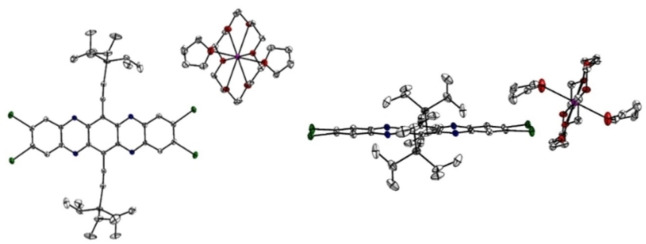
Molecular structure of **I_4_TAP^⋅−^**, the radical anion of **I_4_TAP**, obtained from single‐crystal X‐ray diffraction. Left: front view, right: side view. *P*2_1_/*n*, *a*=11.445(9) Å, *b*=17.953(10) Å, *c*=18.713(12) Å, *α*=90°, *β*=97.68(2)°, *γ*=90° at 100 K.

**Figure 4:**
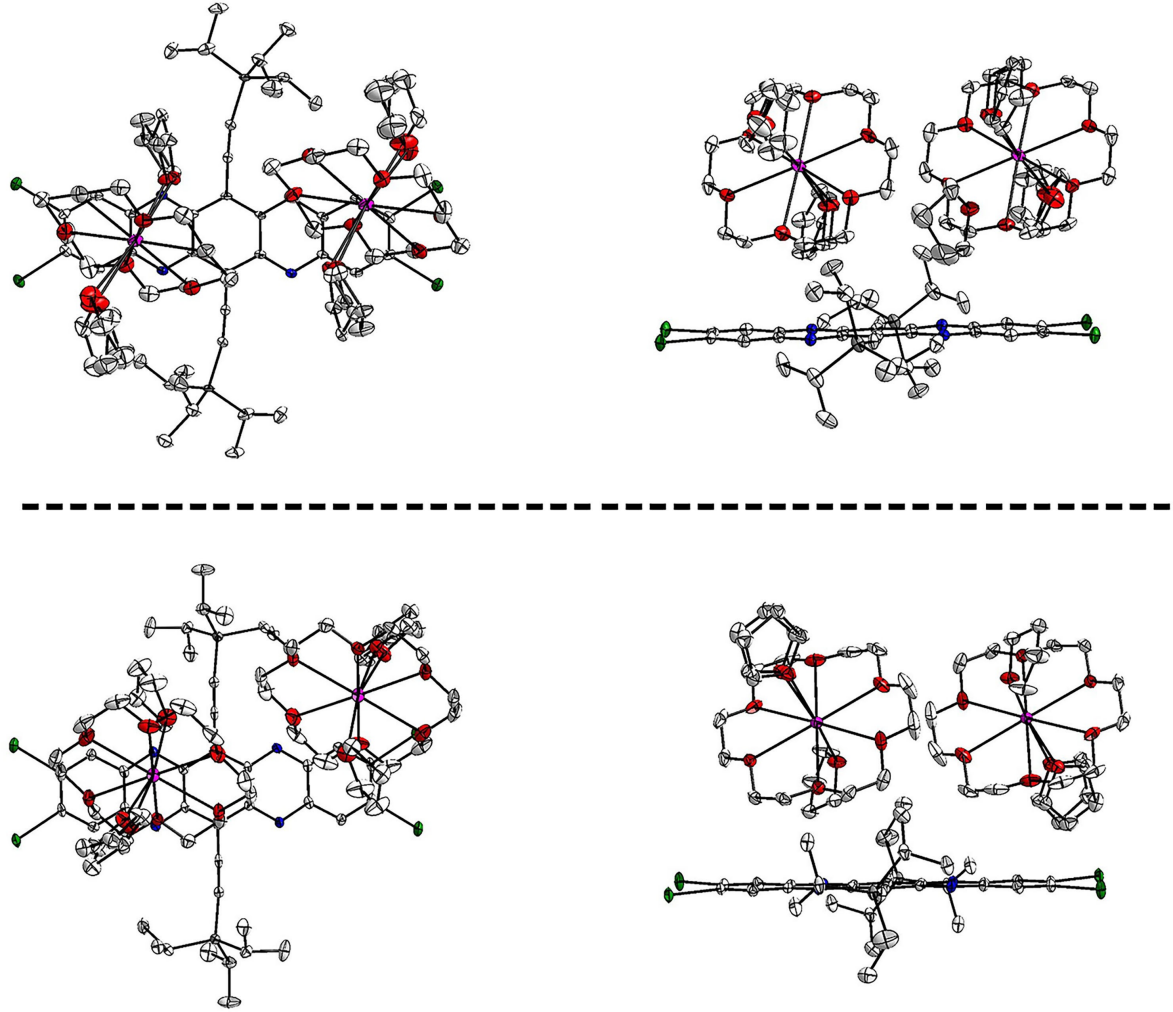
Molecular structures of **I_4_TAP^2−^** polymorphs obtained from single‐crystal X‐ray diffraction. Top: Polymorph B: *P* $\bar{1}$ , *a*=12.926(7) Å, *b*=15.995(7) Å, *c*=16.425(7) Å, *α*=63.903(12)°, *β*=69.29(2)°, *γ*=67.28(2)° at 100 K; bottom: Polymorph A: *P* $\bar{1}$ , *a*=12.5861(13) Å, *b*=14.3108(15) Å, *c*=15.1946(15) Å, *α*=100.426(2)°, *β*=113.678(2)°, *γ*=91.053(2)° at 100 K. Pentane (polymorph A) and THF (polymorph B) solvent molecules were omitted for clarity.

**Figure 5:**
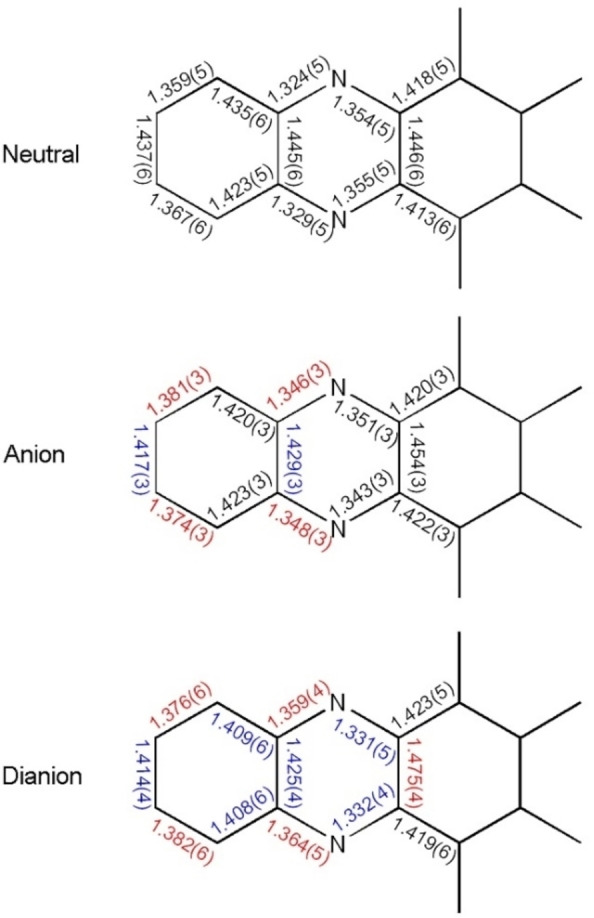
Selected bond lengths (Å) in neutral (top, structure B), radical anion (middle) and dianion (bottom, polymorph B) of **I_4_TAP** (depicted without substituents). Bond lengths in blue for shortening and in red for lengthening compared to the neutral state.

The crystal structure of **I_4_TAP^⋅−^** is displayed in Figure 3. For the dianion **I_4_TAP** ^2−^, we observe two different polymorphs in which the azaacene displays similar geometries (Figure 4). Upon reduction, the acene scaffold remains planar, while the alkyne moieties bend in a slight S‐shape in **I_4_TAP^⋅−^**, and more severely in polymorph B of **I_4_TAP^2−^** (Figure 4, top), while in the second polymorph, the alkynes are linear (Figure 4, bottom). The potassium counterions are isolated from the acene and do not interact with the large π‐system. Consequently, potassium anthracenide does not lead to iodine‐metal exchange, nor is the charge particularly localized on the pyrazine rings, as the differences in the C−N bond lengths are small when comparing neutral and doubly charged **I_4_TAP** (Figure 5). Similar to **Br_4_TAP**, the bond length alternation in the peripheral rings becomes less pronounced when reducing **I_4_TAP** to the radical anion. The trend continues upon further reduction. This behavior was also reported for structurally unrelated compounds.^[20]^ The absolute difference in bond length upon reduction to the radical anion is comparable to that observed for **Br_4_TAP** but considerably higher than observed for **H_4_TAP**.[^10^, ^21^] The C−I bond lengths are unaffected.

**Figure 6:**
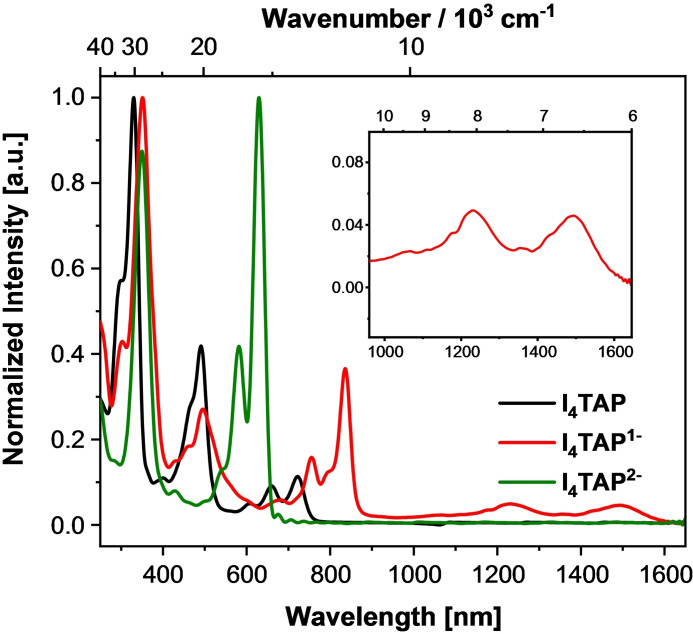
UV‐Vis‐NIR absorption spectra of **I_4_TAP** in its neutral (black), monoanionic (red), and dianionic forms (green). The inset displays the long wavelength absorption region of mono‐reduced **I_4_TAP**.

Monoreduction red‐shifts *λ*_max_ from 720 nm to 1493 nm while *λ*_max_ of the dianion is hypsochromically shifted to 630 nm (Figure 6). These spectroscopic properties strongly resemble those of the tetrabromide. This is also mirrored by the faint fluorescence of **I_4_TAP^2−^** (*λ*_max,em_=713 nm, Supporting Information, Figures S24 and S25). Cyclic voltammetry and spectro‐electrochemistry indicate reduction potentials *E* ^0/−^ of −0.59 V and *E* ^−/2−^ of −1.02 V. **I_4_TAP** is thus more easily reduced than its **Br_4_TAP** analogue (E^0/−^: −0.70 V and E^−/2−^: −1.18 V) despite bromine being a more electronegative substituent. The calculated spectra of neutral **I_4_TAP** and its anions fit the experimental ones (Table 1, Supporting Information, Figure S24). The longest wavelength transitions are all of almost pure HOMO‐LUMO‐character. Mixing of the neutral and the dianionic compound would be expected to lead to comproportionation, based on their calculated stabilities.

**Table 1:** Computed relative energies (Δ*E*) and electronic transitions of **I_4_TAP**, its mono‐ **I_4_TAP^⋅−^**, and dianion **I_4_TAP** ^2**−**^ at the DFT(TDDFT)/ωb97X−D/def2‐TZVPD level of theory.

	**I_4_TAP**	**I_4_TAP** ^***⋅*** **−**^	**I_4_TAP^2−^**
*ΔE* ^*[a]*^	0.00	−4.11	−7.63
*BS_1_* ^*[b]*^	1.89 (0.26) [658 nm]	0.97 (0.10) [1283 nm]	2.36 (1.55) [526 nm]
*Major MO contribution*	H→L 97.8 %	Hβ→Lβ 96.2 %	H→L 95.8 %
*BS_2_* ^*[b]*^	2.99 (1.28) [414 nm]	1.86 (0.50) [668 nm]	
*Major MO Contribution*	H‐1→L 79.4 %	Hα→Lα 92.6 %	

[a] Relative energies in eV. The energy of neutral **I_4_TAP** is set to zero. [b] Vertical excitation energies of low‐lying bright states (BS) in eV, oscillator strengths in parentheses, absorption wavelengths in brackets.

**Figure 7:**
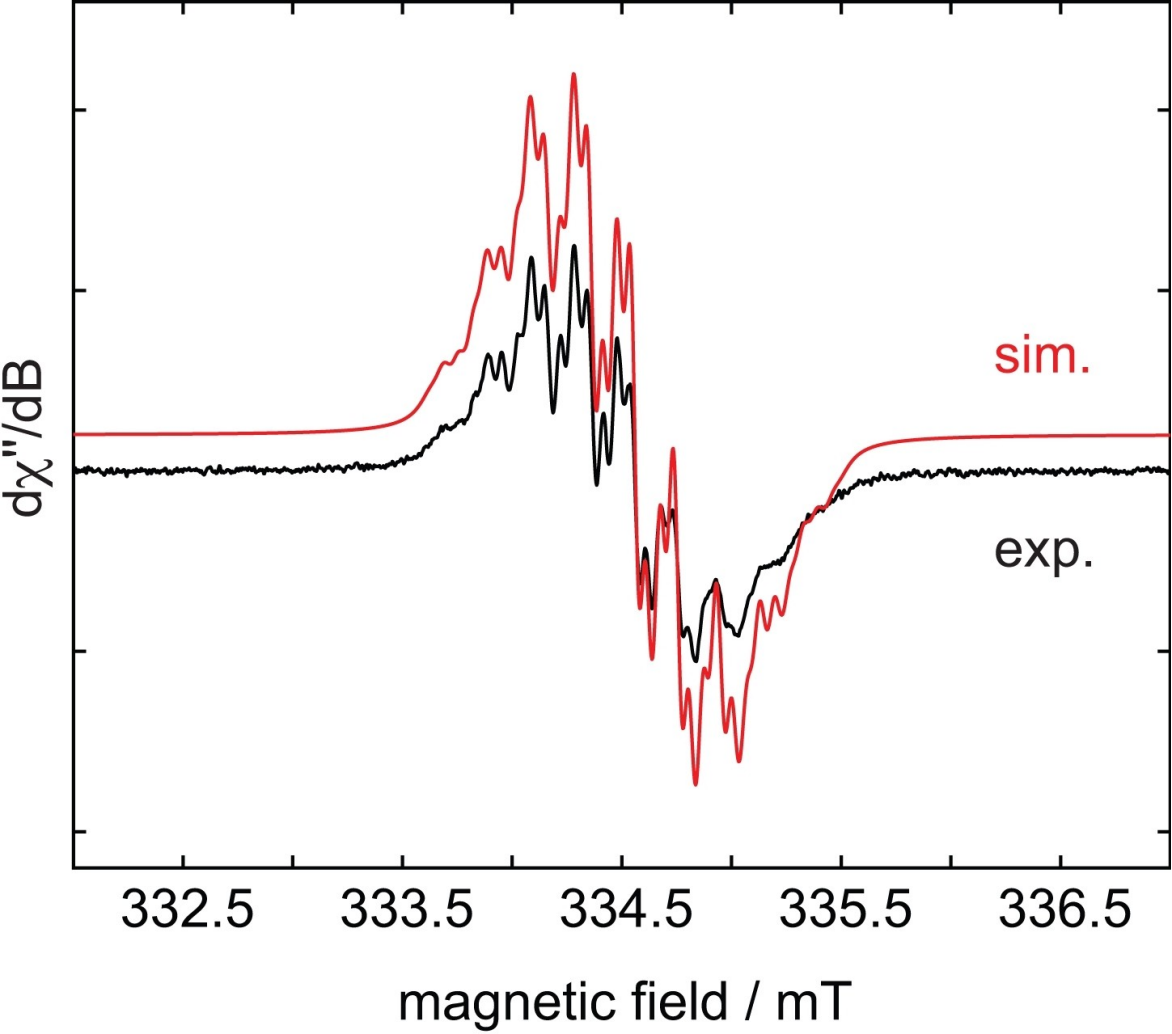
Experimental (black) and simulated (red) continuous‐wave (CW) X‐band EPR spectra of [**I_4_TAP**]^**⋅−**^ in toluene at room temperature. Experimental parameters: microwave frequency=9.38 GHz; microwave power=1 mW; modulation amplitude=0.5 G; conversion time=20 ms; modulation frequency=100 kHz. Simulation parameters: *g*_iso_=2.0037, *a*(^14^N)=5.5 MHz (2.0 G, 4 N) and *a*(^1^H)=1.8 MHz (0.7 G, 4H).

The radical anion of **I_4_TAP** is stable in air and was investigated by electron paramagnetic resonance (EPR) spectroscopy (Figure 7). It displays a multi‐line signal in toluene solution with a g value of 2.0037. An analysis of the EPR spectrum gave the following isotropic hyperfine couplings: *a*(^14^N)=5.5 MHz and *a*(^1^H)=1.8 MHz. The dianion did not show EPR activity, demonstrating its closed‐shell nature. As was the case for the bromine atoms in **Br_4_TAP**,^[10]^ the iodine atoms were also expected to delocalize the spin density more to the outer rings compared to **TAP**. However, based on the very similar nitrogen hyperfine couplings of **I_4_TAP** to **TAP** (a(^14^N)=5.5 MHz),^[14]^ such a situation is not indicated, though one should not overinterpret these data. Due to only partially resolved hyperfine couplings, accurate simulation of the EPR spectra is challenging even in combination with DFT calculations. Frontier molecular orbitals (Supporting Information, Figure S23) and atomic natural charge analyses (Supporting Information, Table S3) reveal that the charge population of the **I_4_TAP** species resembles that of **Br_4_TAP** species. In particular, the natural charges of the terminal rings in **I_4_TAP^⋅−^**, **Br_4_TAP^⋅−^** and **H_4_TAP^⋅−^** indicate a pronounced shift of the negative charge towards the outer rings induced by iodine and bromine substituents.

**Figure 8:**
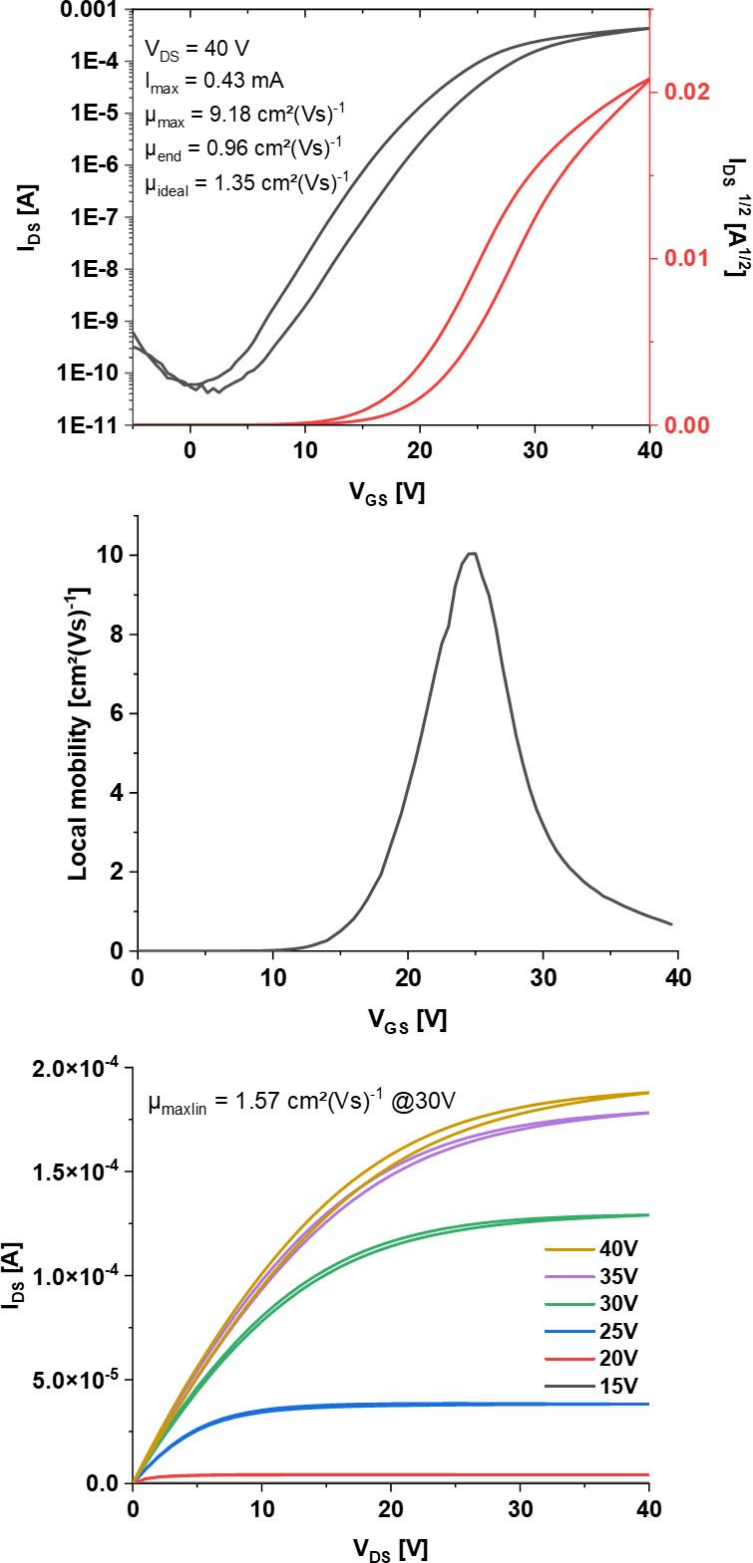
Transfer measurement (top), transfer local mobility plot (middle), and output measurement (bottom) of a top‐performing channel of **I_4_TAP** (*W*=1.26 mm, *L*=83 μm).

Bottom gate/top contact OFETs (Figure S7) were fabricated with silver contact electrodes and a bilayer dielectric consisting of dry SiO_2_ and aluminum oxide coated with a phosphonic acid‐based SAM to prevent trap states.[^7a^, ^22^] The best performing devices were obtained from drop‐cast thin‐films (DCM:acetone 99 : 1 or 95 : 5, 0.5 mg/mL; the screening is described in the “devices” section of the Supporting Information). An acetone content higher than 5 % led to loss of mobility. Transfer curves exhibit “double‐slope” behavior as commonly observed for n‐type semiconductors^[23]^ as well as moderate hysteresis (Figure 8). Such behavior may be related to, among others,^[24]^ contact resistances,^[25]^ charge trapping or differing packing at the surface of the dielectric.^[26]^ Recent literature on mobility overestimation^[27]^ suggests that maximum values extracted from these curves are indicative but not ultimately correct (see Supporting Information, device section) – we followed the proposed guidelines for mobility extraction in such cases. Conservatively extracted mobility values from transfer measurements of 30 channels measured over 7 different substrates gave average electron mobilities of 0.62±0.34 cm^2^(Vs)^−1^ and maximum electron mobilities of up to 1.35 cm^2^(Vs)^−1^. To ensure comparability with **Cl_4_TAP**, maximum mobilities extracted according to procedures used for **Cl_4_TAP** where as high as 9.18 cm^2^(Vs)^−1^. Linear mobilities extracted from output measurements were generally lower than the maximum mobility by factors of 2–5 and dependent on the applied gate voltage.

In comparison with transistor characterization of **Cl_4_TAP** conducted by Miao et al. (same device architecture),^[11]^ **I_4_TAP** displayed lower overall mobilities. This can be rationalized by the poor film morphology of **I_4_TAP**. While XRD measurements show overall 2D brick‐wall morphology in thin‐films and a parallel orientation of the aromatic backbones perpendicular to the substrate with the silyl substituents oriented towards the SAM (see Figure S22), they also contain microscopic pin‐holes as shown via atomic force microscopy (AFM, see Figures S15–S17). Thermal annealing or ageing of the thin‐films under solvent atmospheres at room temperature or fabricating devices using dip‐coating did not resolve this issue.

## Conclusion

In conclusion, we synthesized and characterized **I_4_TAP** as well as its mono‐ and dianion. **I_4_TAP** is a performant, high electron affinity n‐type semiconductor with local electron mobilities as high as 9.18 cm^2^(Vs)^−1^ using Miao's composite dielectric. **I_4_TAP** benefits from brickwall packing in thin‐films dominated by iodine‐iodine interactions (although in single crystals the bulk staircase‐type packing was the dominant crystal form). As the radical anion that carries the charge is air‐stable and not easily re‐oxidized, it avoids trap states. The radical anion shows only small changes in bond lengths when compared to the neutral species. This, most likely, results in low reorganization energies. Further optimization of thin‐film transistors by employing different processing methods such as doctor‐blading or zone‐casting^7b^ could avoid pinhole formation and improve the microstructure in thin‐films resulting in transport properties similar or maybe even superior to that of **Cl_4_TAP**.

## Experimental Section

**Synthesis of I_4_TAP**: Compound **3** (250 mg, 304 μmol, 1.00 equiv.) and 4,5‐diiodocyclohexa‐3,5‐diene‐1,2‐dione (1.10 g, 3.04 mmol, 10.0 equiv.) were reacted in CH_2_Cl_2_:AcOH (1 : 1) at −5 °C for 15 h until TLC (petroleum ether/CH_2_Cl_2_=2 : 1) showed complete conversion of the diamine. The mixture was poured into deionized water and extracted with CH_2_Cl_2_. The combined organic layers were washed with a saturated aqueous sodium bicarbonate solution and subsequently dried over magnesium sulfate. After evaporation of the solvent under reduced pressure, the crude product was purified by chromatography on silica using a gradient of petroleum ether/CH_2_Cl_2_ 4 : 1 ‐>2:1 as eluents to give the crude product as a mixture of the reduced and the oxidized product. This crude mixture was then treated with excess MnO_2_ in CH_2_Cl_2_ for 0.5 h, filtered and the solvent evaporated. The product was obtained as a dark green crystalline solid (209 mg, 182 μmol, 60 %). M.p.: 358 °C. ^1^H NMR (600 MHz, CD_2_Cl_2_): *δ* [ppm]=8.88 (s, 4H), 1.36–1.33 (m, 42H). ^13^C {^1^H} NMR (150 MHz, CD_2_Cl_2_): *δ* [ppm]=144.8, 143.6, 140.6, 123.9, 114.7, 114.2, 103.1, 19.1, 12.0. IR (neat): *ν* [cm^−1^]=2937, 2886, 2859, 1459, 1426, 1357, 1300, 1241, 1120, 1024, 921, 868, 745, 657, 589, 404. HRMS (DART+): *m*/*z* calcd. for C_40_H_46_I_4_N_4_Si_2_: 1146.9512; found: 1146.9510. UV‐Vis: *λ*_max_ (hexane)=727 nm.

**Preparation of [K(18‐crown‐6)(THF)_2_]^+^I_4_TAP^⋅−^**: **I_4_TAP** (5.5 mg, 4.8 μmol) and [K(18‐crown‐6)(THF)_2_] naphthalenide^[15a]^ (2.9 mg, 5.1 μmol) were dissolved in 0.9 mL of dry THF and the mixture was stirred for 10 min to generate a brown solution. The solution was then transferred to four 1 mL GC vials, into which dry pentane vapor was diffused at −30 °C. Dark crystals suitable for single‐crystal X‐ray diffraction formed after 1 week.

**Preparation of [K(18‐crown‐6)(THF)_2_]** ^**+**^_**2**_ **I_4_TAP^2−^**: **I_4_TAP** (5.5 mg, 4.8 μmol) and [K(18‐crown‐6)(THF)_2_] naphthalenide^[15a]^ (6.2 mg, 10.9 μmol) were dissolved in 1.5 mL of dry THF and the mixture was stirred for 15 min to generate a blue solution. Crystals of the α‐form of **I_4_TAP^2−^** were grown by transferring the solution into four 1 mL GC vials, into which dry pentane vapor was diffused at −30 °C. Crystals of the β‐form of **I_4_TAP^2−^** were grown by transferring the solution into four 1 mL GC vials without pentane and storing at −30 °C. Dark crystals suitable for single‐crystal X‐ray diffraction formed after 1 week.

Deposition Number(s) 2155634 (**I**_**4**_ **TAP** 2D brickwall), 2155635 (200 K) and 2133503 (100 K) (**I**_**4**_ **TAP** 1D staircase), 2133504 (**I**_**4**_ **TAP** ^**⋅−**^), 2133505 (polymorph A) and 2133507 (polymorph B) (**I**_**4**_ **TAP** ^**2−**^) contain(s) the supplementary crystallographic data for this paper. These data are provided free of charge by the joint Cambridge Crystallographic Data Centre and Fachinformationszentrum Karlsruhe Access Structures service.

Data related to synthesis and characterization of the neutral compound as well as transistor data are available through heiDATA, the institutional research data repository of Heidelberg University, under [https://doi.org/10.11588/data/S0KS2I].

## Conflict of interest

The authors declare no conflict of interest.

1

## Supporting information

As a service to our authors and readers, this journal provides supporting information supplied by the authors. Such materials are peer reviewed and may be re‐organized for online delivery, but are not copy‐edited or typeset. Technical support issues arising from supporting information (other than missing files) should be addressed to the authors.

Supporting Information

## Data Availability

Data will be published in University of Heidelberg′s Data repository “HeiData” once accepted, the doi is given in the manuscript.
