# Supplementary material for: The Radical Anion, Dianion and Electron Transport Properties of Tetraiodotetraazapentacene
Source: Chemistry. 2022 Oct 25;28(69):e202201919. doi: 10.1002/chem.202201919 (PMC10092590; doi:10.1002/chem.202201919)
Supplement: Supplementary file 1 — Supporting Information [file CHEM-28-0-s001.pdf]

Chemistry–A European Journal

Supporting Information

The Radical Anion, Dianion and Electron Transport Properties of Tetraiodotetraazapentacene

Thomas Wiesner, Zhu Wu, Jie Han, Lei Ji, Alexandra Friedrich, Ivo Krummenacher, Michael Moos, Christoph Lambert, Holger Braunschweig, Benjamin Rudin, Hilmar Reiss, Olena Tverskoy, Frank Rominger, Andreas Dreuw,* Todd B. Marder,* Jan Freudenberg,* and Uwe H. F. Bunz*

Table of contents

Table of contents 1
1. Synthesis 3
2. NMR Spectra 6
3. Devices 9
4. Film Morphology 14
4.1 Polarized Light Microscopy 14
4.2 Atomic Force Microscopy 15
5. X-ray Diffraction 16
5.1 Crystal Structure of the Neutral Compound 16
5.2 Comparison with Cl_4TAP 18
5.3 X-ray Diffraction of Thin-Films 18
6. Computational investigations 20
6.1 Computational details 20
6.2 Frontier molecular orbitals 20
6.3 Natural Charge Population 21
6.4 Absorption Spectra 21
6.5 Nature of Iodine-Iodine Interactions in Crystals 22
7. Photophysical Study 24
8. Spectroelectrochemical Study 25

9. Stability Study 26
10. Crystallographic Data 27
11. References 33

1. Synthesis

General remarks

All commercially available solvents and chemicals were used as delivered. Column chromatography was performed using silica gel from Sigma Aldrich (particle size: 0.032−0.062 mm). THF was dried using an Innovative Technology Inc. solvent purification system (SPS) and stored over potassium. Pentane was distilled from Li[AlH_4], Et_2O used for absorption spectroscopy was dried on the SPS, and both were stored over NaK alloy in an argon-filled glovebox from Innovative Technology Inc. Other reagents and solvents were obtained from commercial suppliers and used without further purification. All reduction reactions were carried out in an argon-filled glovebox unless otherwise stated. NMR spectra were recorded on Bruker Avance Spectrometers using the specified frequency. Chemical shifts (δ) are given in parts per million (ppm) relative to internal solvent signals.[1] The following abbreviations describe the relevant signal multiplicities: s = singlet, m = multiplet. High-resolution mass spectra (HRMS) were obtained by electrospray ionization (ESI) or direct analysis in real time (DART) experiments. IR spectra were recorded from neat samples of the respective analyte on a Jasco FT/IR-4100 spectrometer. CV measurements were performed on a Metrohm Autolab PGSTAT101. UV-vis-NIR absorption and emission spectra were measured on a Varian Cary 5E UV-vis-NIR spectrophotometer or on an Agilent 8453 diode-array UV-vis spectrophotometer unless otherwise stated. The emission and excitation spectra were recorded using an Edinburgh Instruments FLS 920 spectrophotometer equipped with a double monochromator for both excitation and emission, operating in right angle geometry mode. All solutions used in photophysical measurements had concentrations of ca. 10^{-5} M in Et_2O, except where stated. All absorption and emission spectra were recorded under argon in quartz cuvettes specially equipped with a Young's tap. X-band (9.38 GHz) EPR measurements were carried out at room temperature using a Bruker ELEXSYS E580 CW EPR spectrometer. The CW EPR spectrum was measured using 1 mW microwave power and 0.5 G field modulation at 100 kHz, with a conversion time of 20 ms. The spectral simulation was performed using MATLAB 8.6 and the EasySpin 5.0.18 toolbox. Prime grade P-doped Si-wafers with 100 nm thermally grown silicon dioxide and low resistivity (R < 0.005 Ω) were purchased from Si-Mat. Metals used for device contacts were produced by Kurt J. Lesker Company (East Sussex, United Kingdom) and thermally evaporated. Pictures were taken under ambient conditions on a Nikon Eclipse LV100POL microscope with the included camera.

Single-crystal X-ray diffraction

Crystals suitable for single-crystal X-ray diffraction were selected, coated in perfluoropolyether oil, and mounted on MiTeGen sample holders or microloops. Due to their lack of transparency, the crystal choice was based on its shape. They were mounted under an argon stream and cooled to 100 K, and to 200 K for **I_4TAP** structure B, using an Oxford Cryostream low temperature device attached to the diffractometer. Diffraction data of **I_4TAP** structure B at 200 K, **$I_4TAP^{\bullet-}$**, and **I_4TAP^{2-}** polymorphs A and B were collected on a Bruker X8 Apex II 4-circle diffractometer with a CCD area detector, using Mo-K_α radiation generated by a Nonius FR591 rotating anode or a micro-focus sealed X-ray tube and monochromated by multi-layer focusing mirrors. Diffraction data of **I_4TAP** structure B at 100 K were collected on a Rigaku Oxford Diffraction XTALAB Synergy-S, Dualflex, four-circle diffractometer with a semiconductor HPA-detector (HyPix-6000) and a micro-focus sealed X-ray tube providing multi-layer mirror monochromated Cu-K_α radiation. Diffraction data of **I_4TAP** structure A were collected on a STOE Stadivari four-circle diffractometer with a semiconductor HPA-detector (Dectris Pilatus 300K) and a micro-focus sealed X-ray tube providing multi-layer mirror monochromated Cu-K_α radiation. The images were processed and corrected for Lorentz-polarization effects and absorption (empirical scaling) as implemented in the Bruker software packages, in the CrysAlis^{Pro} software from

Rigaku OD, or in X-Area LANA 1.70.0.0 from STOE, respectively. The structures were solved using the intrinsic phasing method (SHELXT) [2] and Fourier expansion technique. All non-hydrogen atoms were refined in anisotropic approximation, with all hydrogen atoms 'riding' in idealized positions, by full-matrix least squares against F^2 of all data, using SHELXL[3] software and the SHELXLE[4] graphical user interface. Diamond[5] software was used for graphical representation. Crystal data and experimental details are listed in Tables S6-S11; full structural information has been deposited with Cambridge Crystallographic Data Centre. CCDC-2155634 (**I_4TAP** structure A), 2133503 (**I_4TAP** structure B at 100 K), 2155635 (**I_4TAP** structure B at 200 K), 2133504 (**$I_4TAP^{•–}$**), 2133505 (**$I_4TAP^{2–}$** polymorph A), and 2133507 (**$I_4TAP^{2–}$** polymorph B). These data can be obtained free of charge from The Cambridge Crystallographic Data Centre *via* www.ccdc.cam.ac.uk/data_request/cif.

The synthesis was adapted from the PhD thesis of Hilmar Reiss, *Halogenierte TIPS-Tetraazapentacene,* **2019**, Heidelberg, Germany.

Diiodo-*ortho*-quinone

Diiodocatechol[6] (100 mg, 273 µmol, 1.00 equiv.) was dissolved in 10.0 mL CH_2Cl_2 at 0 °C, then sodium periodate (70.9 mg, 332 µmol, 1.20 equiv.) and tetra-*n*-butylammonium bromide (3.56 mg, 11.1 µmol, 0.04 equiv.) in 2.00 mL of deionized water were added. The mixture was stirred for 30 min at this temperature before it was poured into ice cold water. Ice cold CH_2Cl_2 was used for extraction. The combined organic phases were used without further purification before warming to room temperature.

7,8-diiodo-4,11-bis((tri*iso*propylsilyl)ethynyl)-[1,2,5]thiadiazolo[3,4-b]phenazine 2

4,7-bis((triisopropylsilyl)ethynyl)benzo[c][1,2,5]thiadiazole-5,6-diamine **1** (1.00 g, 1.90 mmol, 1.00 equiv.) and diiodo-*ortho*-quinone (1.37 g, 3.80 mmol, 2.00 equiv.) were reacted in CH_2Cl_2 / AcOH (1:1) at −5 °C for 15 h until TLC showed complete conversion of the starting material (petroleum ether / CH_2Cl_2 = 2:1). The mixture was poured into deionized water and extracted with CH_2Cl_2. The organic phases were treated with sat. aqueous sodium bicarbonate solution and dried over sodium sulfate. After evaporation of the solvent, the crude product was purified by chromatography on silica using petroleum ether / CH_2Cl_2 5:1 -> 3:1 as eluent to give the product as a dark green crystalline solid (1.30 g, 1.53 mmol, 80.5%).
M.p.: 276 °C. ^{1}H NMR (400 MHz, $CDCl_3$): δ [ppm] = 8.79 (s, 2H), 1.32 – 1.29 (m, 42H). ^{13}C {^{1}H} NMR (100 MHz, $CDCl_3$): δ [ppm] = 155.3, 144.3, 142.7, 140.3, 115.0, 113.3, 113.0, 102.0, 19.0, 11.7. IR (neat): ν [cm^{-1}] = 2941, 2863, 1438, 1362, 1177, 1018, 881, 866, 804, 734, 675, 635, 570, 500, 407. HR-MS(DART+): m/z calcd. for $C_{34}H_{45}I_2N_4SSi_2$: 851.0987; found: 851.1005. Elemental analysis (%) calcd. for $C_{34}H_{44}I_2N_4SSi_2$: C 48.00, H 5.21, N 6.59, found: C 48.30, H 5.35, N 6.31. UV-Vis: λ_{max} (hexane) = 667 nm.

7,8-diiodo-1,4-bis((tri*iso*propylsilyl)ethynyl)phenazine-2,3-diamine 3

In a heat-gun dried Schlenk tube under an argon atmosphere thiadiazolophenazine **2** (500 mg, 588 µmol, 1.00 equiv.) was dissolved in 25.0 mL dry THF / 625 µL dry MeOH (40:1). At −10 °C, SmI_2 (72.0 mL, 7.20 mmol, 0.1 M in THF, 12.3 equiv.) was added dropwise. After 10 min at this temperature the red mixture was poured into sat. aqueous NaCl solution. Et_2O was used for extraction. The combined organic phases were washed with a sat. aqueous NaS_2O_3

solution and water and then dried over sodium sulfate. After evaporation of the solvent under reduced pressure, the crude product was purified by column chromatography using petroleum ether / ethyl acetate 8:1 as eluent to give the product as an orange solid (475 mg, 578 µmol, 98.3%).
M.p.: 185 °C decomp. ^{1}H NMR (600 MHz, $CDCl_3$): δ [ppm] = 8.66 (s, 2H), 4.95 (s, 4H), 1.25 – 1.23 (m, 42H). ^{13}C {^{1}H} NMR (150 MHz, $CDCl_3$): δ [ppm] = 144.5, 141.78, 141.5, 139.6, 107.0, 104.8, 102.3, 100.3, 19.0, 11.5. IR (neat): ν [cm^{-1}] = 2941, 2863, 1438, 1362, 1177, 1018, 881, 866, 804, 734, 675, 635, 570, 500, 407. HR-MS(ESI+): m/z calcd. for $C_{34}H_{49}I_2N_4Si_2$: 823.1580; found: 823.1582.

2,3,9,10-tetraiodo-6,13-bis((tri*iso*propylsilyl)ethynyl)quinoxalino[2,3-b]phenazine I_4TAP

Compound **3** (250 mg, 304 µmol, 1.00 equiv.) and 4,5-diiodocyclohexa-3,5-diene-1,2-dione (1.10 g, 3.04 mmol, 10.0 equiv.) were reacted in CH_2Cl_2:AcOH (1:1) at −5 °C for 15 h until TLC (petroleum ether / CH_2Cl_2 = 2:1) showed complete conversion of the diamine. The mixture was poured into deionized water and extracted with CH_2Cl_2. The combined organic layers were washed with a saturated aqueous sodium bicarbonate solution and subsequently dried over magnesium sulfate. After evaporation of the solvent under reduced pressure, the crude product was purified by chromatography on silica using a gradient of petroleum ether / CH_2Cl_2 4:1 -> 2:1 as eluents to give the crude product as a mixture of the reduced and the oxidized product. This crude mixture was then treated with excess MnO_2 in CH_2Cl_2 for 0.5 h, filtered and the solvent evaporated. The product was obtained as a dark green crystalline solid (209 mg, 182 µmol, 60%). Mp.: 358 °C. ^{1}H NMR (600 MHz, CD_2Cl_2): δ [ppm] = 8.88 (s, 4H), 1.36 – 1.33 (m, 42H). ^{13}C {1H} NMR (150 MHz, CD_2Cl_2): δ [ppm] = 144.8, 143.6, 140.6, 123.9, 114.7, 114.2, 103.1, 19.1, 12.0. IR (neat): ν [cm^{-1}] = 2937, 2886, 2859, 1459, 1426, 1357, 1300, 1241, 1120, 1024, 921, 868, 745, 657, 589, 404. HRMS (DART+): m/z calcd. for $C_{40}H_{46}I_4N_4Si_2$: 1146.9512; found: 1146.9510. UV-Vis: λ_{max} (hexane) = 727 nm.

Synthesis and crystallization of the anion and dianion

Preparation of **$[K(18\text{-crown-6})(THF)_2]^+I_4TAP^{\bullet-}$**

I_4TAP (5.5 mg, 4.8 µmol) and $[K(18\text{-crown-6})(THF)_2]$ naphthalenide[7] (2.9 mg, 5.1 µmol) were dissolved in 0.9 mL of dry THF and the mixture was stirred for 10 min to generate a brown solution. The solution was then transferred to four 1 mL GC vials, into which dry pentane vapor was diffused at -30 °C. Dark crystals suitable for single-crystal X-ray diffraction formed after 1 week.

Preparation of **$[K(18\text{-crown-6})(THF)_2]_2^+I_4TAP^{2-}$**

I_4TAP (5.5 mg, 4.8 µmol) and $[K(18\text{-crown-6})(THF)_2]$ naphthalenide[7] (6.2 mg, 10.9 µmol) were dissolved in 1.5 mL of dry THF and the mixture was stirred for 15 min to generate a blue solution. Crystals of Polymorph A of **I_4TAP^{2-}** were grown by transferring the solution into four 1 mL GC vials, into which dry pentane vapor was diffused at -30 °C. Crystals of Polymorph B of **I_4TAP^{2-}** were grown by transferring the solution into four 1 mL GC vials without pentane and storing at -30 °C. Dark crystals suitable for single-crystal X-ray diffraction formed after 1 week.

2. NMR Spectra

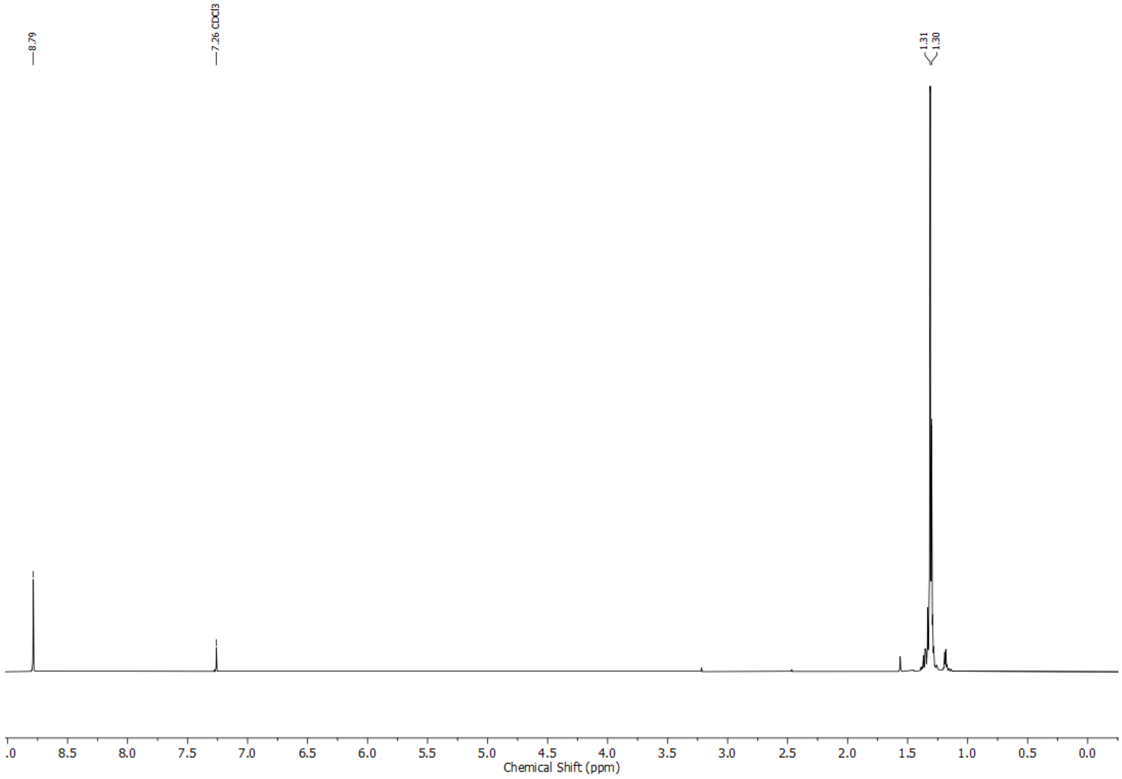

Figure S 1. ^{1}H NMR spectrum (400 MHz, $CDCl_3$) of thiadiazole **2**.

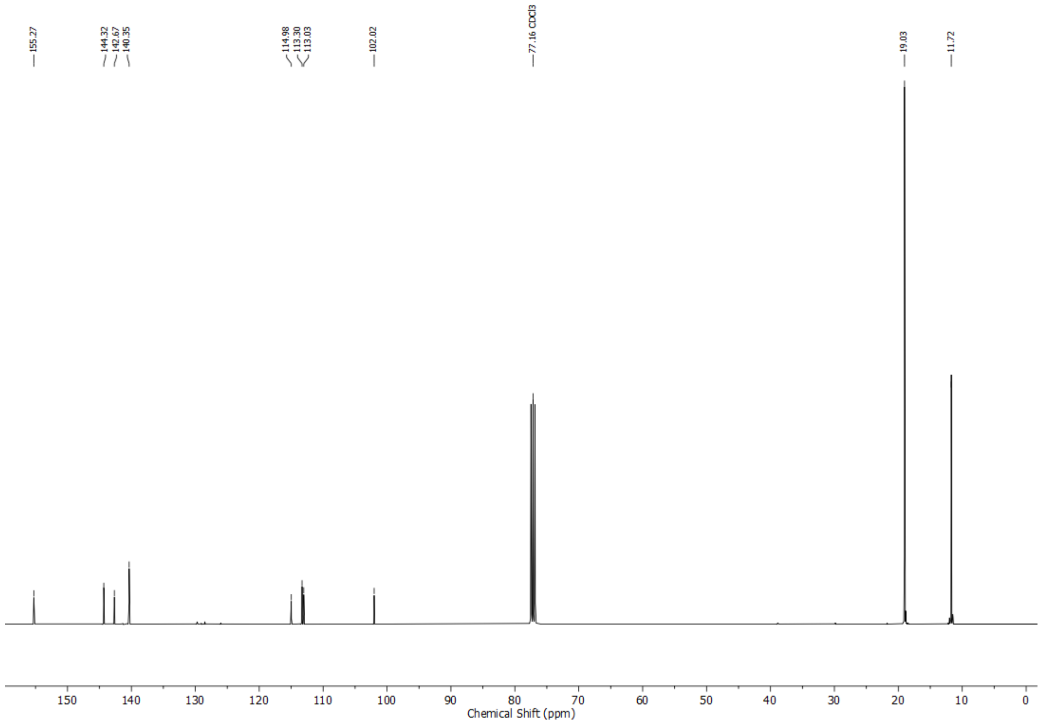

Figure S 2. $^{13}C\{^{1}H\}$ spectrum (101 MHz, $CDCl_3$) of thiadiazole **2**.

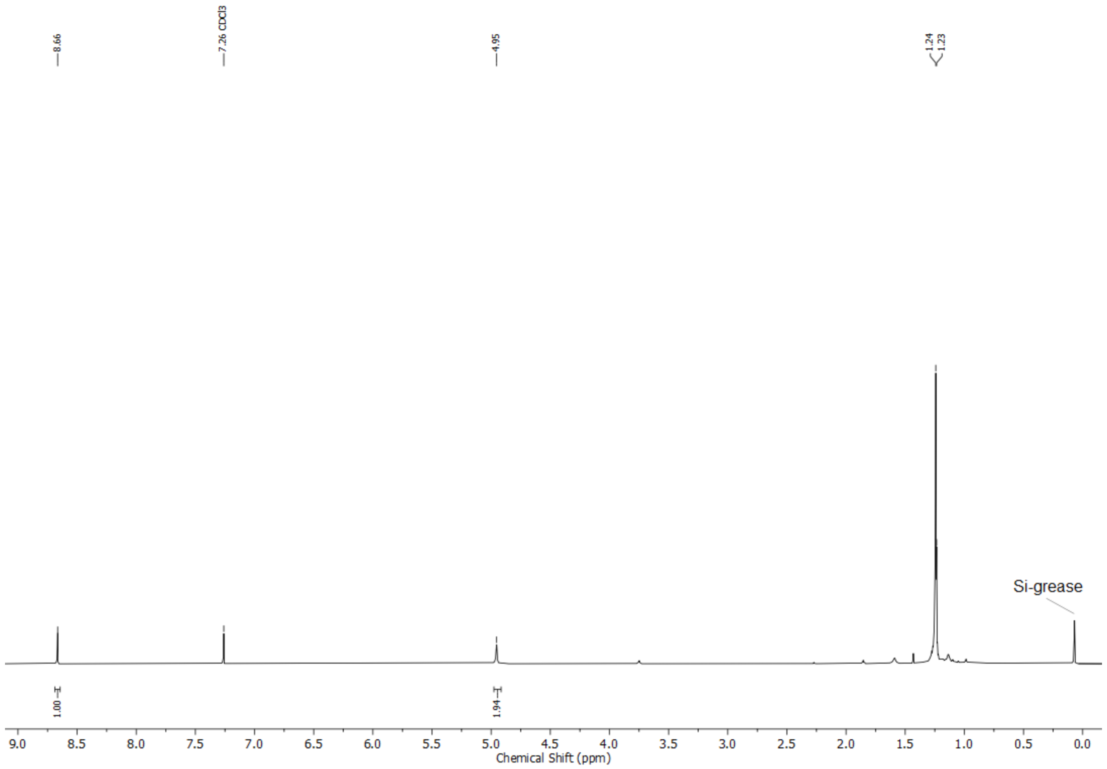

Figure S 3. ^{1}H NMR spectrum (600 MHz, $CDCl_3$) of diamine **3**.

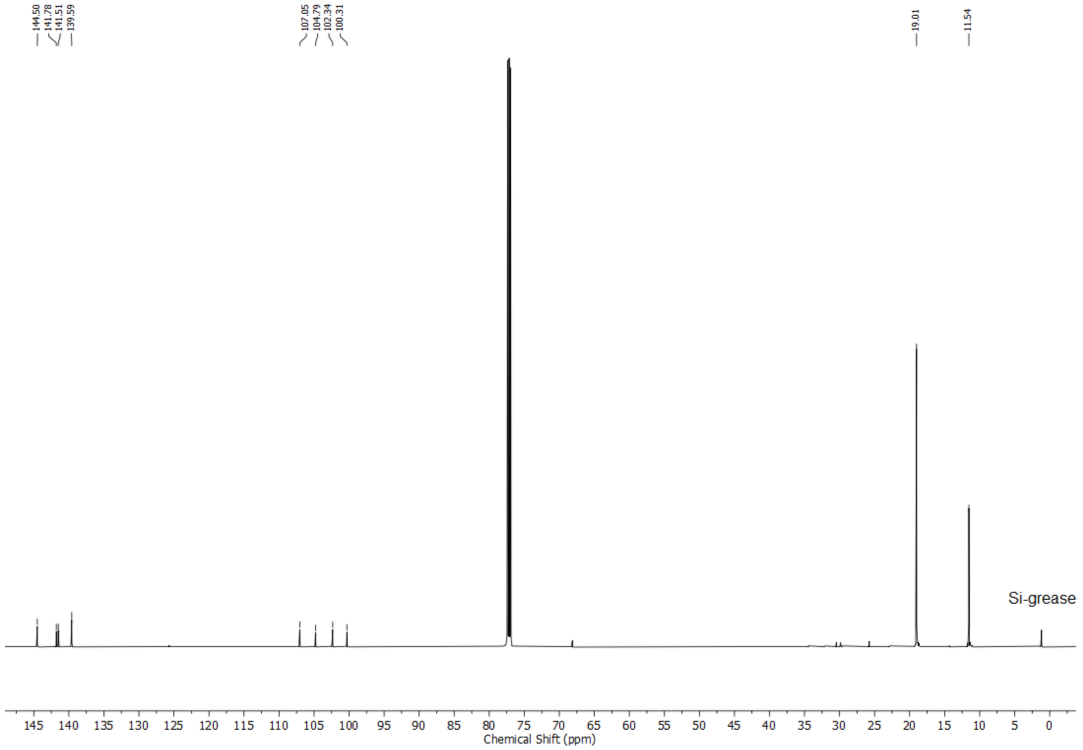

Figure S 4. $^{13}C\{^{1}H\}$ spectrum (150 MHz, $CDCl_3$) of diamine **3**.

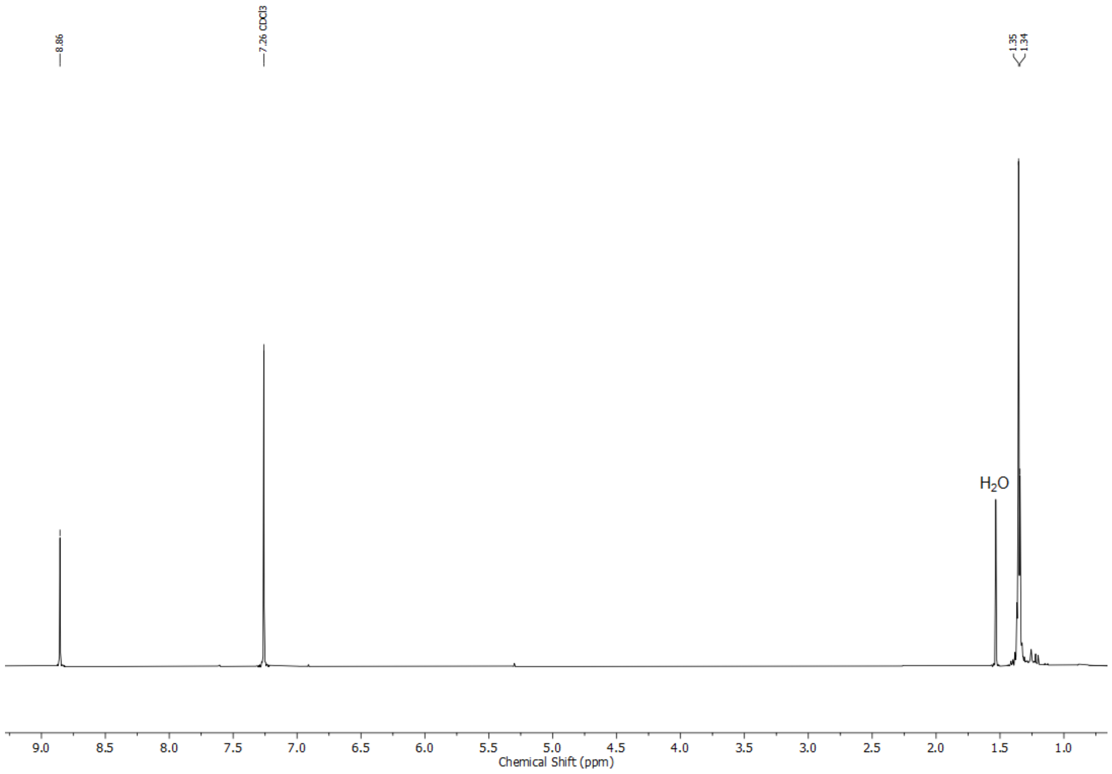

Figure S 5. ^{1}H NMR spectrum (600 MHz, $CDCl_3$) of **I_4TAP**.

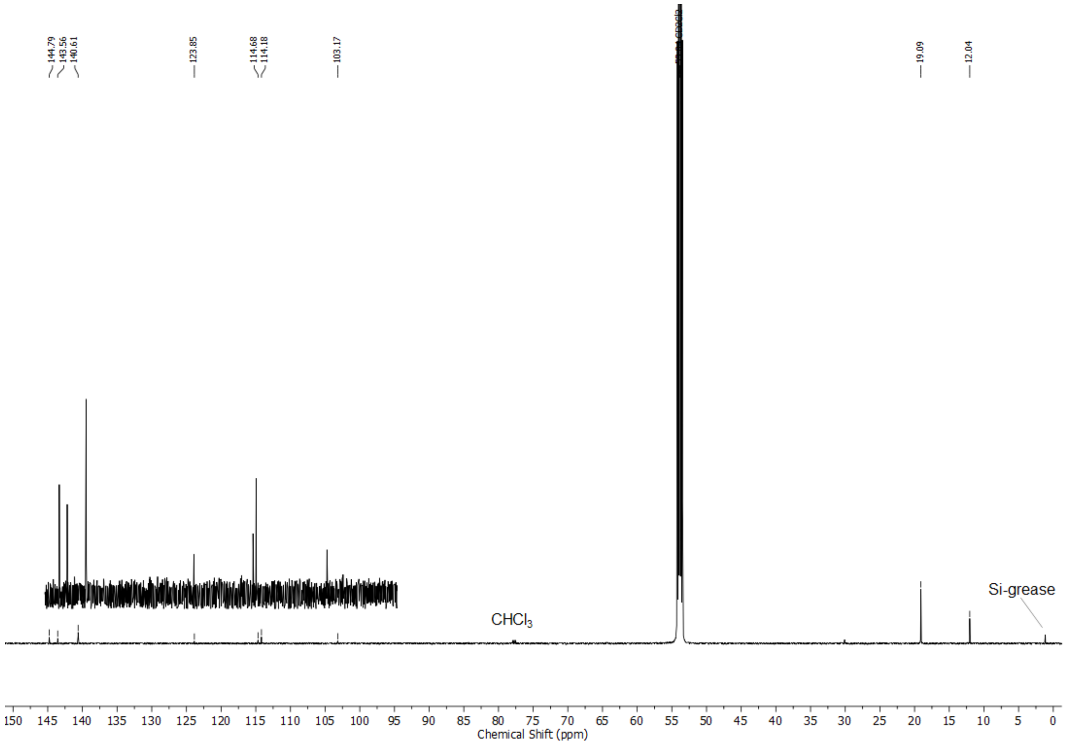

Figure S 6. $^{13}C\{^{1}H\}$ spectrum (150 MHz, CD_2Cl_2) of **I_4TAP**.

3. Devices

Wafer preparation

Deionized water of Millipore quality and HPLC grade solvents were used for wafer preparation and device preparation. **I_4TAP** was purified using silica gel chromatography and recrystallized three times by layering methanol over a solution of **I_4TAP** in CH_2Cl_2 and letting the CH_2Cl_2 evaporate over the course of several days. The crystals were separated and washed with pentane and diethyl ether.

Highly doped Si-wafers (R < 0.005 Ω) with 100 nm thermally grown SiO_2 were subsequently washed for 10 min in an ultrasonic bath with acetone, *iso*propanol and ethanol. Afterwards, the wafers were washed with deionized water and blown dry using a N_2-gun. They were then treated with piranha acid (H_2SO_4/H_2O_2 3:1) at 100 °C for 15 min, rinsed with deionized water, blown dry using a stream of nitrogen and directly spin-coated with a 150 mM solution of $Al(NO_3)_3 \cdot 9\,H_2O$ in ethanol (5000 rpm, 40 s). These wafers were immediately placed on a hot plate and baked at 300 °C for 30 min. The wafers were allowed to cool for 2 min and subsequently put into a 15 mM solution of 12-cyclohexyldodecylphosphonic acid (CDPA)[8] in isopropanol overnight. Residual CDPA was washed off the wafers through ultrasonication in *iso*propanol for 10 min and rinsing with deionized water. The capacitance of the dielectric layer is 26.5 nFcm^{-2}.

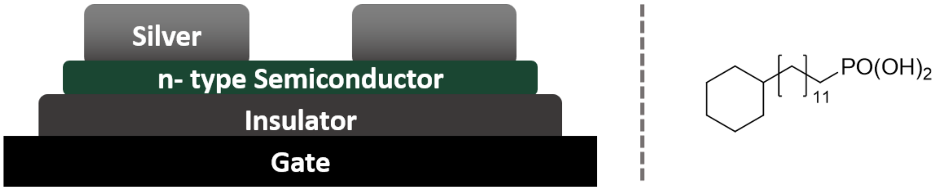

Figure S 7. Left: Device architecture. The insulator consists of 100 nm dry SiO_2, spin-coated Aluminum oxide and CDPA-SAM. Right: Molecular structure of CDPA.

Drop-cast films of **I_4TAP** were prepared by dropping the solution onto the wafer as well as next to the wafer and covering with a lid to ensure slow evaporation and protection from airflow.

Silver contact pads were thermally deposited with a thickness of 40 nm through shadow masks at pressures below 2 x 10^{-6} bar and rates of 0.04 Å/s. Transistor characteristics were measured with a semiconductor characterization system (Keithley 4200-SCS) in a nitrogen filled glove box. A channel was measured if the growth direction of the underlying film was approximately perpendicular to the contact pads (±30 ° as estimated by eye) and the morphology of the film at that position was representative for that wafer. Before measurement, the area of the thin-film around the contact pads was confined by hand under the microscope using a needle. A typical transfer measurement was conducted in dual sweep mode and had a scanning speed of ca. 3 Vs^{-1}. Field effect mobilities were extracted from transfer characteristics using

$$\mu = \left(\frac{\delta I_{DS}^{\frac{1}{2}}}{\delta V_G} \right)^2 \frac{2L}{W C_i}$$

where μ is the field effect mobility, I_{DS} is the source-drain current, V_G is the gate voltage, W is the channel width, L the channel length and C_i is the capacitance per unit area of the gate dielectric layer. The slope of $I_{DS}^{1/2}$ was averaged

over 5 V for every measurement point and the highest value chosen to calculate μ_{max}. The slope of $I_{DS}^{1/2}$ over the last 5 V of a measurement was used to calculate μ_{end}.

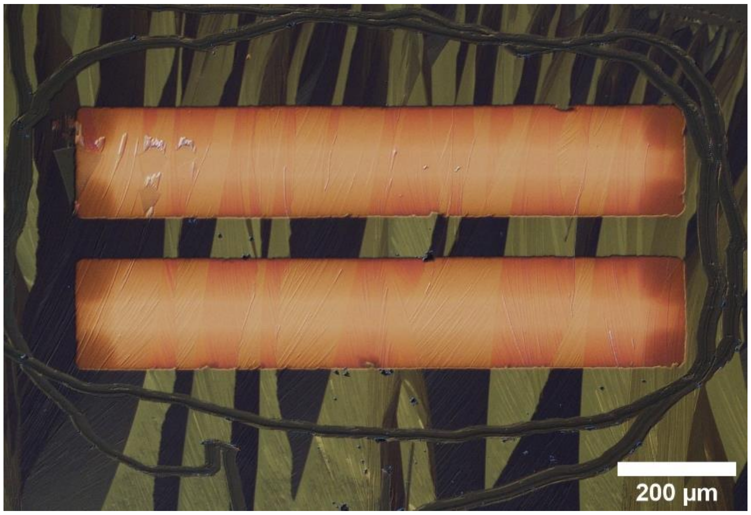

Figure S 8. Silver electrodes on **I$_4$TAP** thin-film were confined by hand under the microscope with a needle.

Screening

The choice of solvent for drop-casting films on was thoroughly investigated. First, pure solvents were tested at concentrations of 0.5 mg mL^{-1}. CH_2Cl_2 gave films resembling those of other **X$_4$TAP**-derivatives and was further investigated.

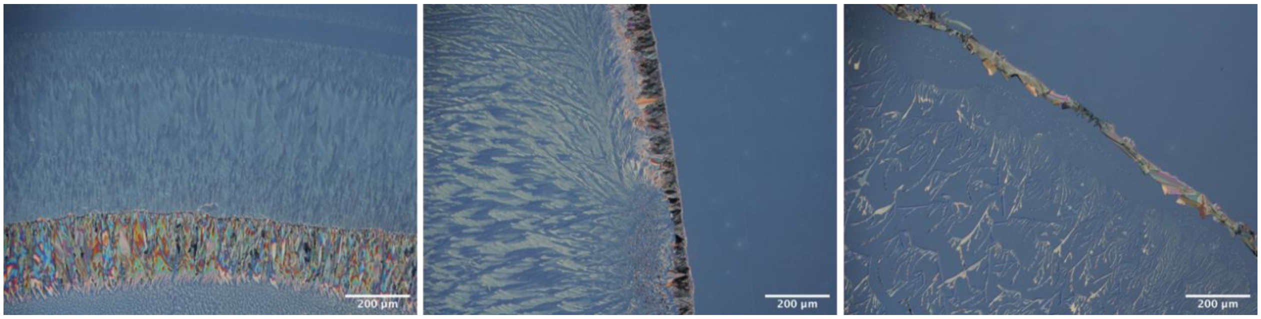

Figure S 9. Thin-films of **I$_4$TAP** drop-cast at room temperature and c = 0.5 mg mL^{-1} under polarized light. Left: Thin-film drop-cast from CH_2Cl_2; Middle: Thin-film drop-cast from $CHCl_3$; Right: Thin-film drop-cast from Toluene at 50 °C.

X$_4$TAP derivatives are usually drop-cast from mixtures of a “good” and a “bad” solvent in respect to the solubility of the **TAP**.[9] Different additives were tested for suitability. As **I$_4$TAP** was not soluble in the typical 1:1 ratio, a ratio of 95:5 of CH_2Cl_2 to acetone, acetonitrile or ethyl acetate were tested.

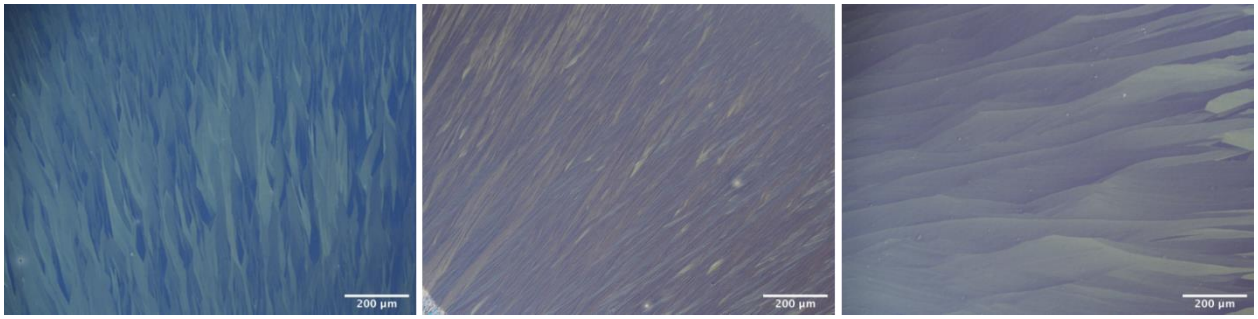

Figure S 10. Thin-films of **I₄TAP** drop-cast at room temperature and c = 0.5 mg mL^{-1} under polarized light. Left: Thin-film drop-cast from CH_2Cl_2:acetone 95:5; Middle: Thin-film drop-cast from CH_2Cl_2:MeCN 95:5; Right: Thin-film drop-cast from CH_2Cl_2:ethyl acetate 95:5.

All solvent systems exhibit better film formation than pure CH_2Cl_2 in regards to grain size and film area. Investigating the device performance of transistors fabricated from these films showed that CH_2Cl_2:acetone performed best by far (Table S 1). Maximum mobility μ_{max} was estimated using a local slope over a range of 5 V, μ_{end} takes into account the last measuring points before reaching $V_{g,max}$. Further testing showed that higher concentrations of acetone were detrimental to the device performance, while lowering the concentration had no significant effect. At this stage, no effort was undertaken to reach statistical significance.

Table S 1. Saturation regime mobility of best performing transistors using the respective solvent ratio for drop-casting.

Solvent	μ_{max} [cm^2(Vs)$^{-1}$]
CH_2Cl_2	1.71
CH_2Cl_2:acetone 95:5	9.27
CH_2Cl_2:MeCN 95:5	3.02
CH_2Cl_2:ethyl acetate 95:5	1.11

Solvent	μ_{max} [cm^2(Vs)$^{-1}$]
CH_2Cl_2:acetone 9:1	4.57
CH_2Cl_2:acetone 95:5	9.27
CH_2Cl_2:acetone 99:1	9.18

Nearly all curves showed double-slope behavior. As Podzorov *et al.* state, this oftentimes leads to charge carrier mobility overestimation.[10] To give additional context to our results, the ideality factor *r* proposed by Podzorov *et al.* was determined for all measurements, whereas

$$r_{max/end} = \frac{\mu_{ideal}}{\mu_{max/end}}$$

and

$$\mu_{ideal} = \left(\frac{\sqrt{I_{DS,max}} - \sqrt{I_{DS}(V_G = 0)}}{V_{G,max}}\right)^2 \frac{2L}{WC_i}$$

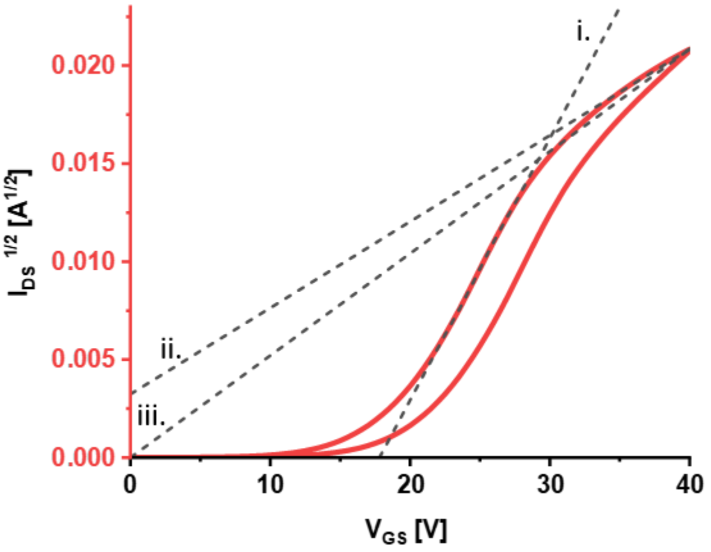

Figure S 11. Different slopes used to calculate transfer mobilities. i) μ_{max} uses maximum slope over 5 V during the measurement; ii) μ_{end} uses the slope over the last 5 V of the measurement; iii) μ_{ideal} uses the slope between $I_{DS}^{1/2}(V_G = 0\ V)$ and $I_{DS}^{1/2}(V_{G,max} = 40\ V)$.

Due to the insignificant differences between 1% and 5% acetone in CH_2Cl_2, both ratios were further investigated. For transfer measurements of 1% acetone in CH_2Cl_2, an average of 2.13 ± 1.82 cm² $(Vs)^{-1}$ with a median of 1.60 cm²$(Vs)^{-1}$ and average reliability factor of 54% ± 26% could be found for 30 channels over 7 wafers. Linear mobility values extracted from output-measurements showed lower mobility values by factors of 2-5, but still higher than μ_{ideal} extracted from transfer measurements. Using 5% acetone in CH_2Cl_2, the average was 2.29 ± 1.75 cm² $(Vs)^{-1}$ with a median of 1.92 cm² $(Vs)^{-1}$ and an average reliability factor of 41% ± 22% for 35 channels over 5 wafers. Linear mobility values extracted from output-measurements showed lower mobility values by a factor of 2-5, and generally lower than μ_{ideal} extracted from transfer measurements. A summary including the differently extracted transfer mobilities and statistical indicators can be found in Table S 2. These results support the hypothesis that there is no significant difference between the two solvent systems.

Table S 2. Summary of transfer measurements conducted with 1% and 5% acetone in CH_2Cl_2 at concentrations of 0.5 mg mL^{-1}, respectively. µ is given in cm²$(Vs)^{-1}$.

	**1% acetone in CH_2Cl_2 30 channels over 7 wafers**	**5% acetone in CH_2Cl_2 35 channels over 5 wafers**
**Max μ_{max}**	9.18	9.27
**Ø μ_{max}**	2.13 ± 1.82	2.29 ± 1.75
**Median μ_{max}**	1.60	1.92
**Ø Ideality factor μ_{max}**	54% ± 26%	41% ± 22%
**Median ideality factor μ_{max}**	56%	35%
**Max μ_{ideal}**	1.35	1.45
**Ø μ_{ideal}**	0.62 ± 0.34	0.56 ± 0.30
**Median μ_{ideal}**	0.53	0.60
**Max μ_{end}**	1.99	1.69
**Ø μ_{end}**	0.90 ± 0.40	0.71
**Median μ_{end}**	0.88	0.74
**Ø Ideality factor μ_{end}**	79% ± 40%	86% ± 48%
**Median ideality factor μ_{end}**	63%	72%

Additionally, dip-coating was studied as a second mode of thin-film generation. As a solvent system, CH_2Cl_2:acetone 95:5 was chosen and optimized for concentration and pulling speed.

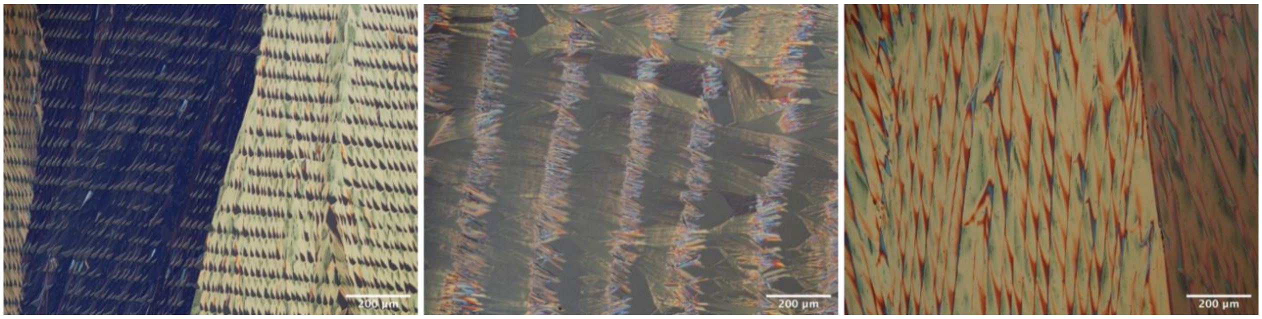

Figure S 12**.** Thin-films of **I_4TAP** in CH_2Cl_2:acetone 95:5 fabricated using dip-coating at a pulling speed of 0.3 mm min^{-1} using different concentrations. Left: 0.5 mg mL^{-1}; Middle: 1.0 mg mL^{-1}; Right: 1.5 mg mL^{-1}.

Concentration optimization shows that a concentration higher than 1.0 mg mL^{-1} is necessary to obtain films with homogenous areas of film. Consequently, 1.5 mg mL^{-1} was chosen as a concentration with which the pulling speed was optimized. As there were no significant changes in morphology, devices were fabricated and the mobilities compared.

Table S 3. Maximum mobilities extracted from transfer measurements measured for dip-coated films of **I_4TAP** at c = 1.5 mg mL^{-1} at different pulling speeds.

**Pulling speed [mm min^{-1}]**	**Max. μ_{max} [cm^2 (Vs)$^{-1}$]**
0.30	1.89
0.40	3.91
0.50	4.46
0.60	4.25

For 0.5 mm min^{-1}, a peak in mobility was found. The lower maximum value that was obtained leads to the conclusion that dip-coating thin-films are more homogenous, but exhibit lower film quality.

4. Film Morphology

4.1 Polarized Light Microscopy

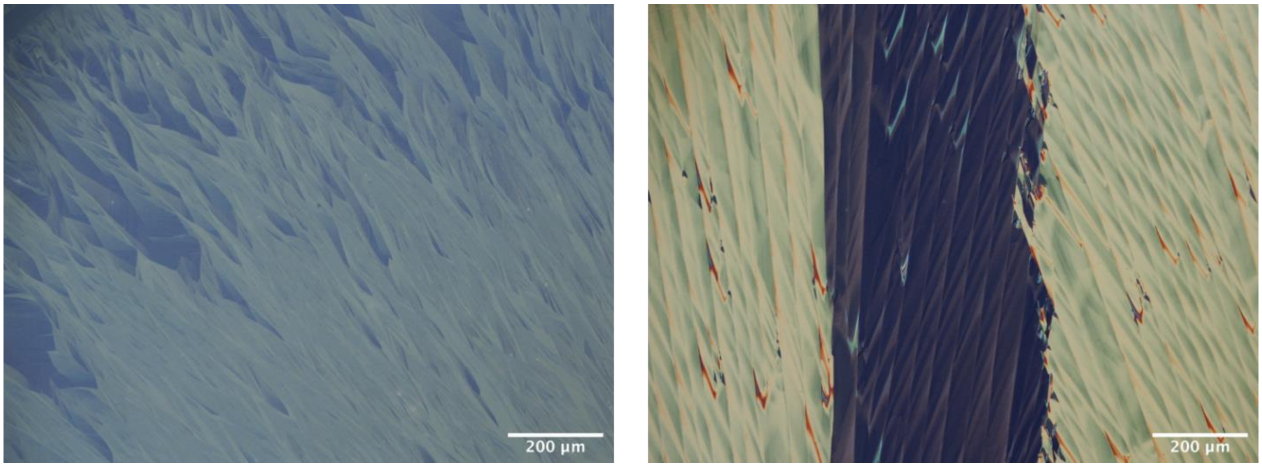

Figure S 13. Left: Drop-cast film of **I_4TAP** from 0.5 mg mL^{-1} solution in CH_2Cl_2:acetone 95:5. Right: Dip-coated film of **I_4TAP** from 1.5 mg mL^{-1} solution in CH_2Cl_2:acetone 95:5 at a pull-up speed of 0.5 mm min^{-1}.

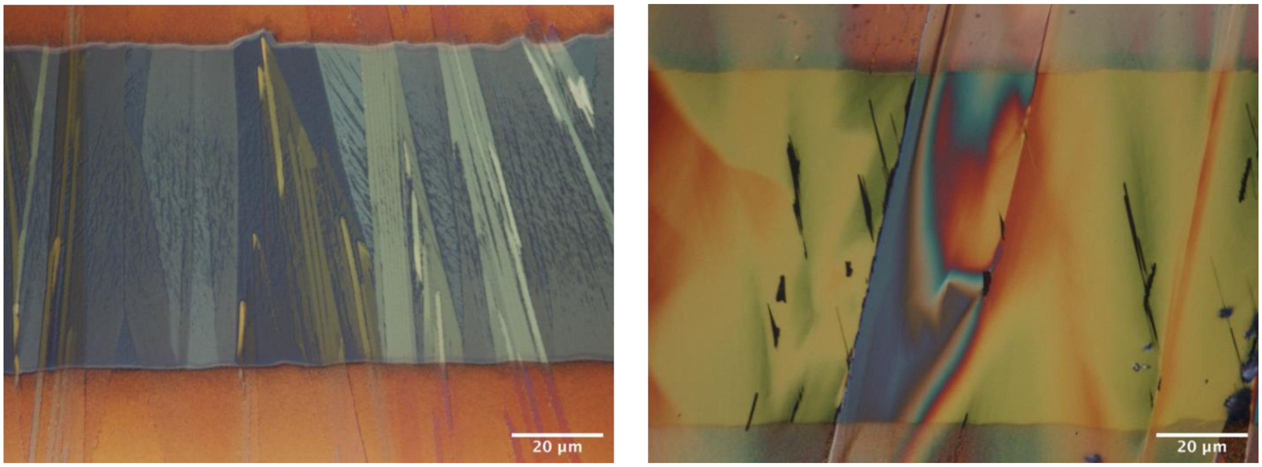

Figure S 14. **I_4TAP** thin-films after aging in a saturated CH_2Cl_2 atmosphere at room temperature for 1 h. Left: Channel with drop-cast film from 0.5 mg mL^{-1} solution in CH_2Cl_2:acetone 99:1. Right: Channel with dip-coated film of **I_4TAP** from 1.5 mg mL^{-1} solution in CH_2Cl_2:acetone 95:5 at a pull-up speed of 0.5 mm min^{-1}.

4.2 Atomic Force Microscopy

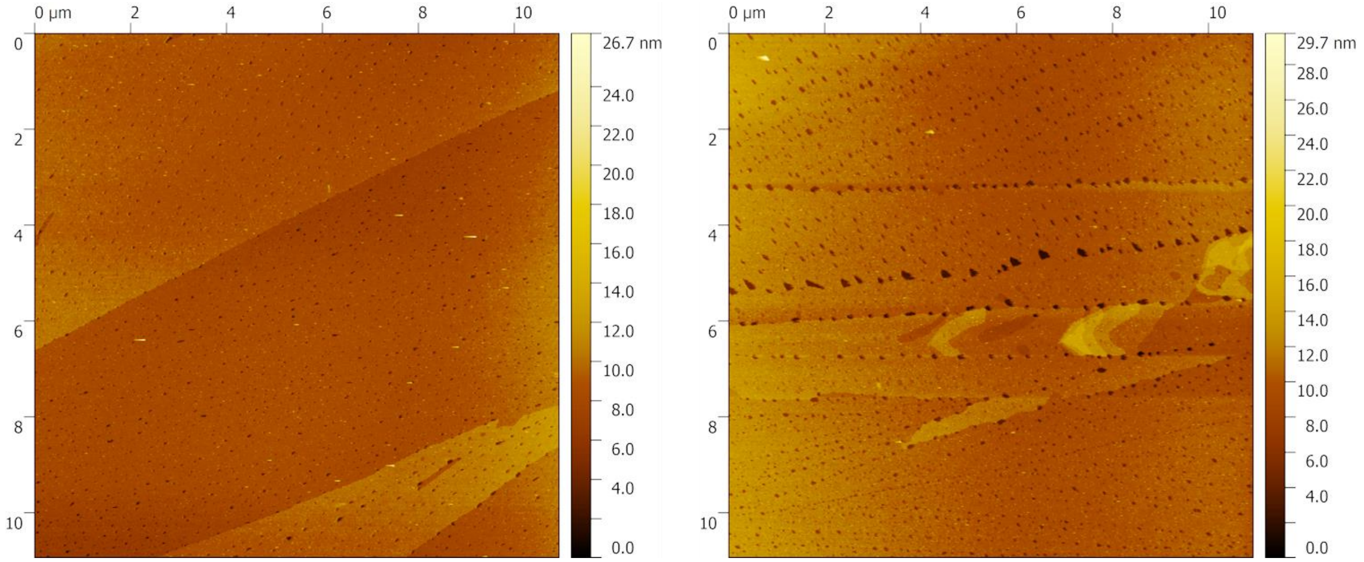

Figure S 15. AFM images of drop-cast films of **I₄TAP** from 0.5 mg mL^{-1} solution in CH_2Cl_2:acetone 95:5 before (left) and after (right) thermal annealing at 100 °C for 1 h in a nitrogen filled glovebox. Pinholes appeared unchanged or larger. Charge carrier mobilities diminished by 85-99% were measured after treatment.

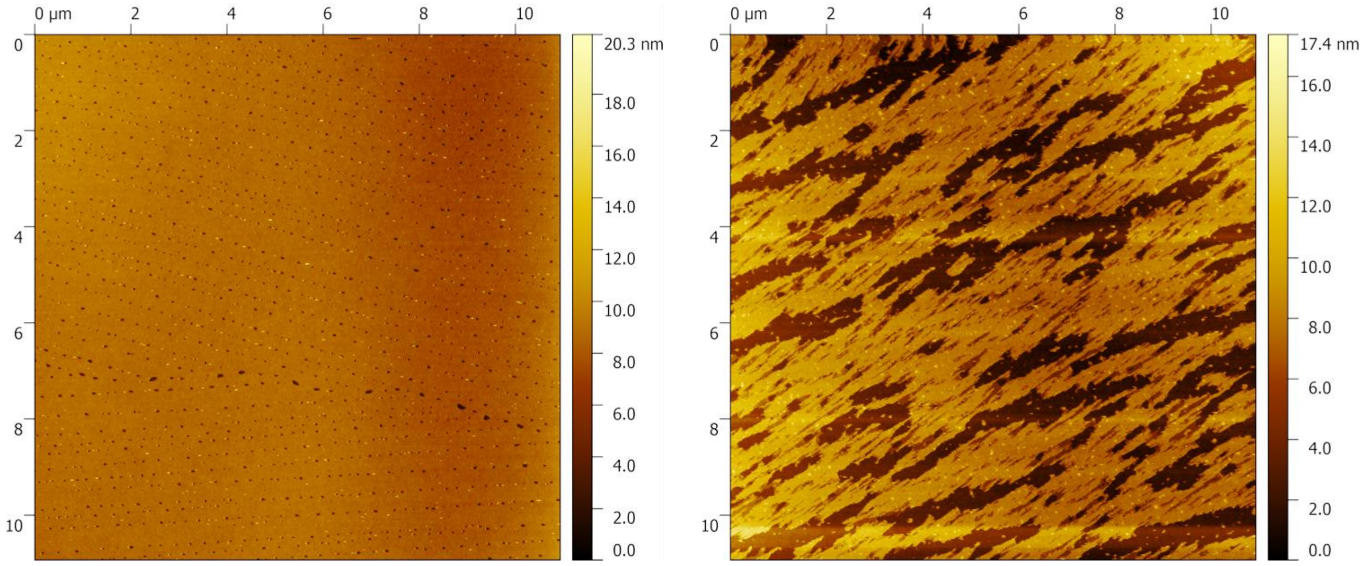

Figure S 16. AFM images of drop-cast films of **I₄TAP** from 0.5 mg mL^{-1} solution in CH_2Cl_2:acetone 99:1 before (left) and after (right) aging in a saturated CH_2Cl_2 atmosphere at room temperature for 1 h. The thin-film disintegrates, no charge carrier mobilities could be measured after treatment.

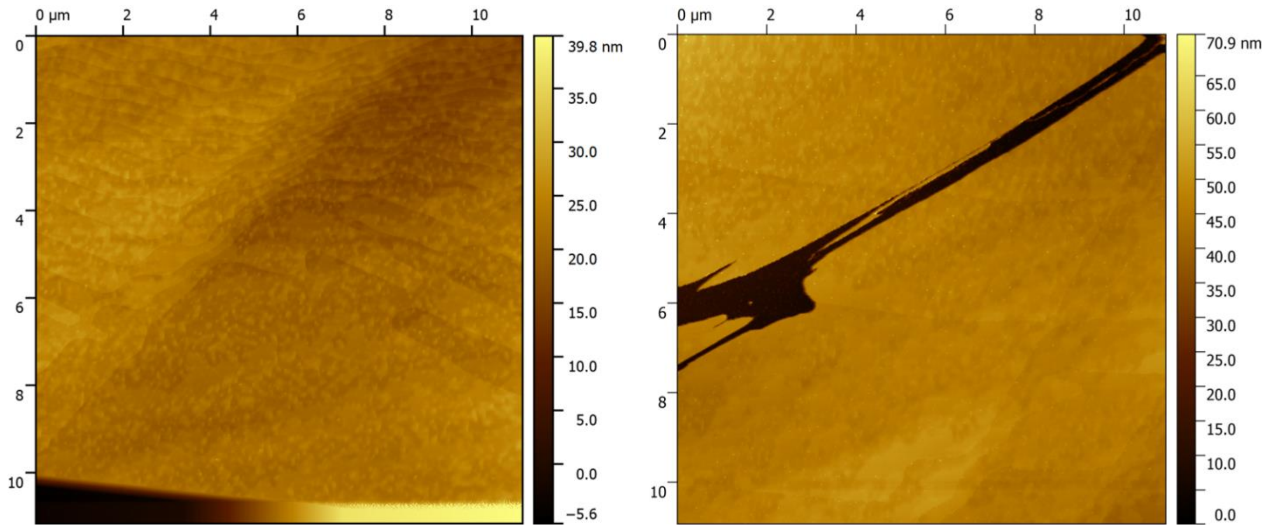

Figure S 17. AFM images of dip-coated films of **I₄TAP** from 1.5 mg mL^{-1} solution in CH_2Cl_2:acetone 95:5 before (left) and after (right) aging in a saturated CH_2Cl_2 atmosphere at room temperature for 1 h. The dip-coated film displays a rough texture. After treatment, large cavities appeared in the film. No charge carrier mobilities could be measured.

5. X-ray Diffraction

5.1 Crystal Structure of the Neutral Compound

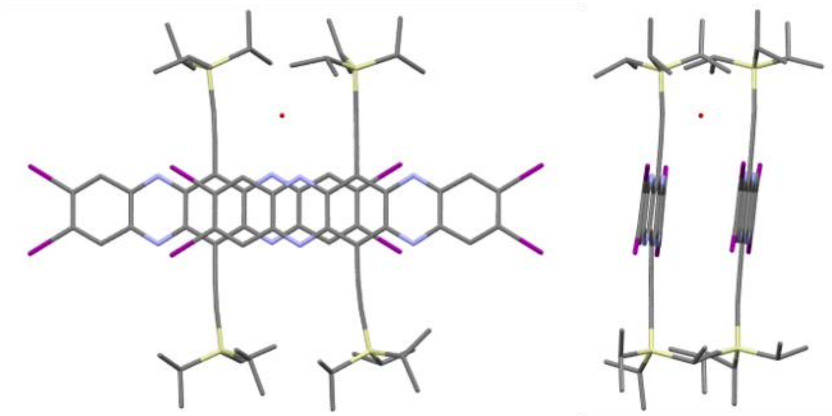

Figure S 18. Molecular structures of the two symmetry-independent molecules of **I$_4$TAP** structure A and crystal water (left: Front view; right: Side view).

Reproducing the formation of structure A was attempted at various temperatures (70 °C, 20 °C, 7 °C, -20 °C) from various solvents ($CHCl_3$ mixed with petrol ether, ethyl acetate, acetone, methanol) using regular grade solvents and also with an added drop of water, to no avail.

Neutral struct A
100 K

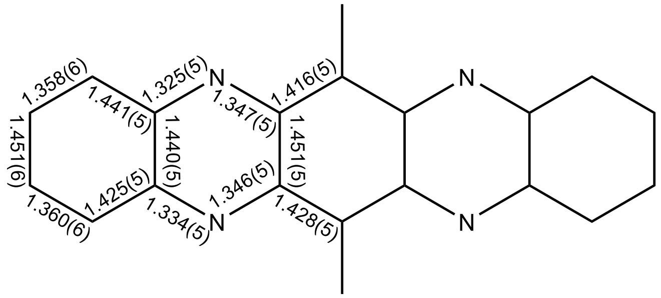

Neutral struct A
100 K

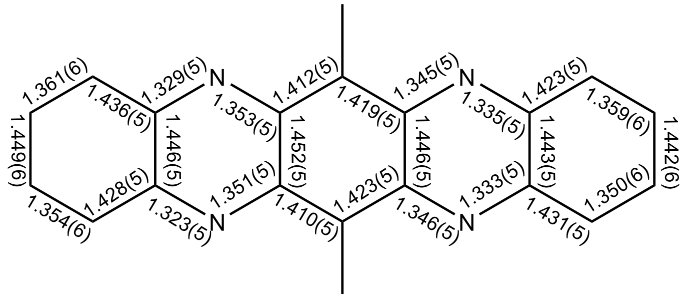

Neutral struct B
100 K

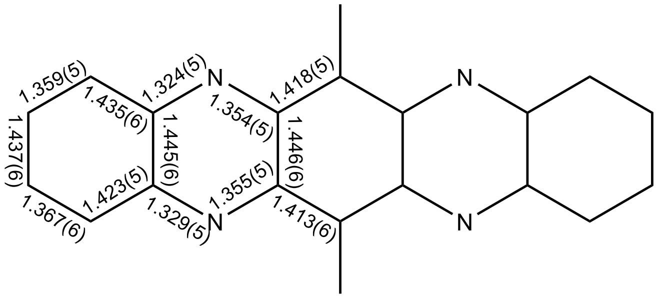

Neutral struct B
200 K

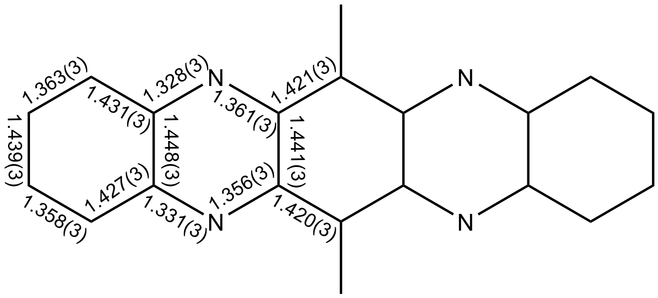

Figure S 19. Bond lengths of the core of **I_4TAP** in its neutral form. Top: P structure A (Brickwall) at 100 K with two independent molecules in its unit cell, one of which is centrosymmetric. Bottom: structure B (Staircase) at 100 and 200 K with one centrosymmetric molecule in its respective unit cell.

5.2 Comparison with Cl_4TAP

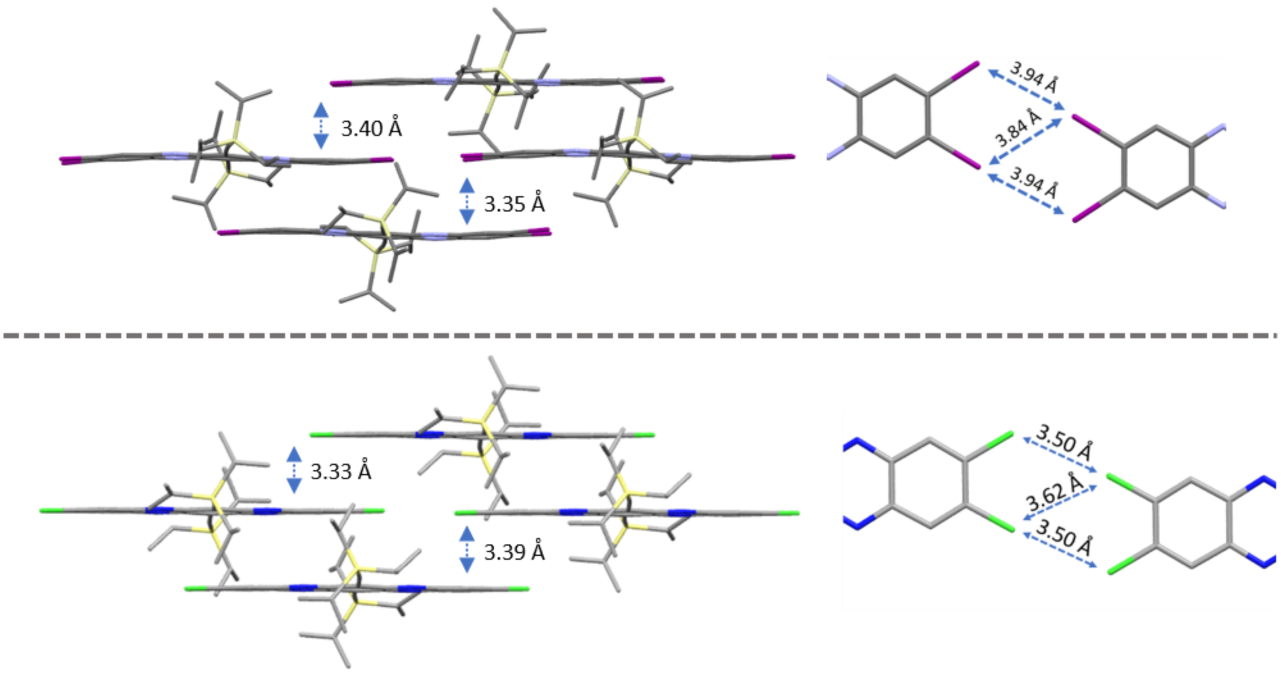

Figure S 20. Halogen-halogen-distance in solid-state-packing of **Cl_4TAP** (CCDC: XIGLUX (1845318), see ref. 9a).

The van-der-Waals radius of chlorine is 1.75 Å, that of iodine 1.98 Å. The Cl-Cl-distances in **Cl_4TAP** add up to twice the vdW-distance of Cl, not suggesting strong interactions as opposed to **I_4TAP**. The lateral offset between the dimer pairs (right) is more elaborate for **I_4TAP** than for **Cl_4TAP**. This is evident from the decrease in the "diagonal" I-I distance for **I_4TAP** (3.84 Å vs. 3.94 Å) as compared to **Cl_4TAP**, where the corresponding distance is larger (3.62 Å vs. 3.50 Å).

5.3 X-ray Diffraction of Thin-Films

Measurements of **I_4TAP** thin-films drop-cast on CDPA-modified surfaces from 0.5 mg mL^{-1} solutions in CH_2Cl_2:acetone 95:5 show that the thin-films exhibit the brickwall motif (Figure S 21).

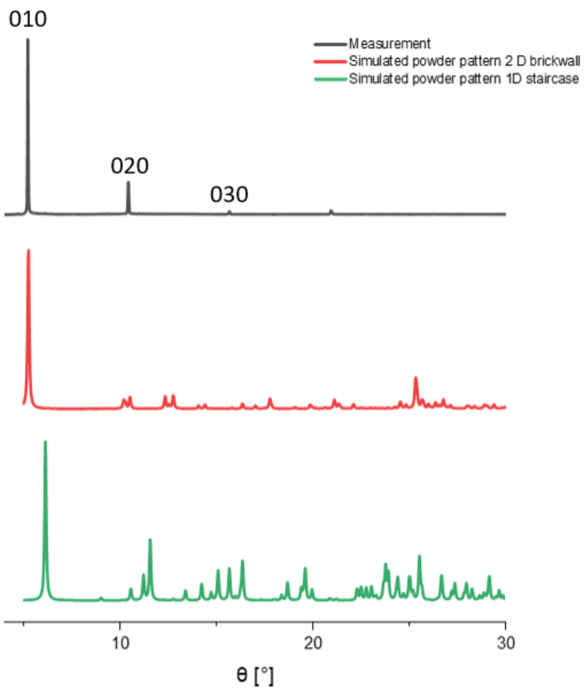

Figure S 21. Surface X-ray diffraction data of thin-films drop-cast from CH_2Cl_2:acetone 95:5 compared to simulated powder patterns for both structures of **I_4TAP** showing that 2D brickwall packing is the packing mode for well-performing **I_4TAP** devices.

The peaks correspond to the crystal lattice plane (010), meaning that this plane is oriented in parallel to the surface. Measuring the angle between that lattice plane and the π-plane of **I_4TAP** shows that the molecules contact the surface with their tri*iso*propylsilyl-moieties and stand upright with an angle of 89.21 ° (Figure S 22). This means the conducting channel lies in parallel to the surface, which is considered to be favorable.

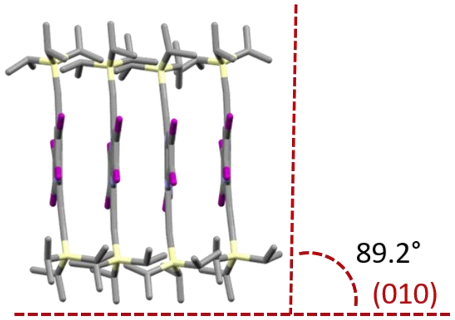

Figure S 22. Assessment of the angle in which the molecules are oriented with respect to the surface.

6. Computational investigations

6.1 Computational details

Geometries of the neutral, anionic, and dianionic species of **I_4TAP** were optimized with DFT using the range-separated exchange-correlation functional ωB97X-D[11] and the def2-SVP basis set.[12] Energies and natural charge population of the optimized structures were calculated with the def2-TZVPD basis set.[13] The vertical excited states were computed at the theoretical level of TDDFT[14]/ωB97X-D/def2-TZVPD. THF solvation effects were incorporated by the polarizable continuum model using the integral equation formalism variant (IEFPCM).[15] For comparison, natural charge population of **H_4TAP** and **Br_4TAP** was conducted at the same level of theory as **I_4TAP**. To study the iodine-iodine interactions in crystals, binding energies (BE) were computed for dimers taken from structures A and B of **I_4TAP** (namely DM-A and DM-B) at ωB97X-D/def2-TZVP level of theory. Basis set superposition error (BSSE) was corrected through the counterpoise approach.[16] Based on the wavefunctions obtained from the single point calculations of the relative monomers and dimers, molecular interaction (independent gradient model based on Hirshfeld partition, IGMH)[17] electrostatic potential (ESP)[18] and atom-in-molecules (AIM)[19] analyses were performed by Multiwfn 3.9 version[20] and visualized by VMD[21] programs. To better understand the interaction nature, symmetry-adapted perturbation theory (SAPT)[22] calculations were conducted at the scaled SAPT0/def2-TZVP level of theory with PSI4 1.6.1 code.[23] The -TIPS side groups were replaced by H atoms to reduce the computational cost of the SAPT calculations. All DFT calculations have been performed using Gaussian 16 Rev. C.01.[24]

6.2 Frontier molecular orbitals

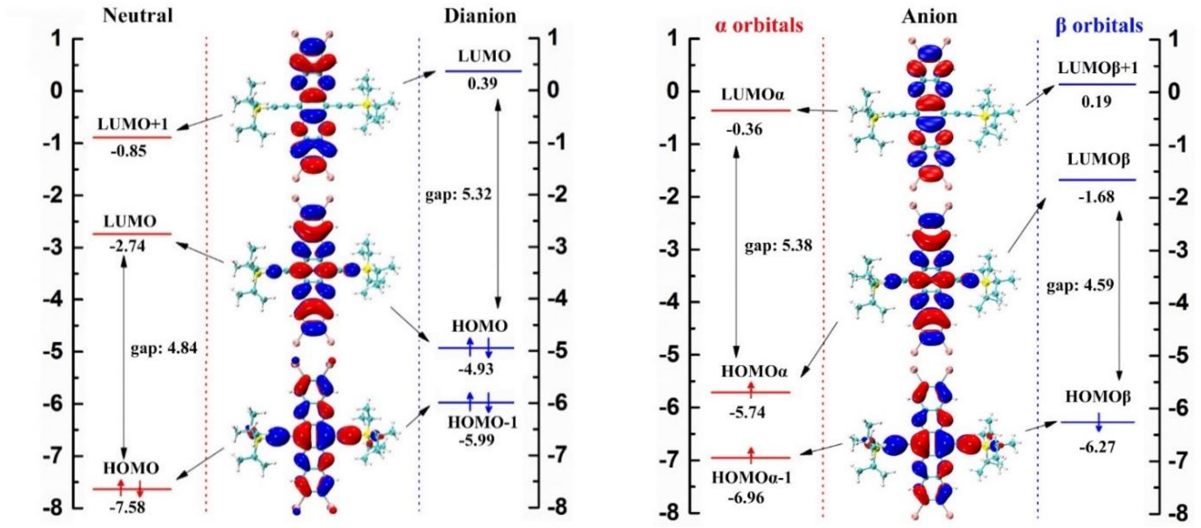

Figure S 23. Kohn–Sham frontier molecular orbital diagrams and energies (in eV) for neutral, dianionic (left) and anionic **I_4TAP** (right) (isodensity value: ±0.02).

6.3 Natural Charge Population

Table S 4. Atomic natural charges of neutral, anionic and dianionic **H_4TAP**, **Br_4TAP** and **I_4TAP**. The charges of each ring are summed according to the numbering scheme on the right, and the TIPS groups are also included in ring **3**. Charges of atoms shared by two rings are split. The charge population of **I_4TAP** species is very similar to that of **Br_4TAP** species. The negative charges are shifted to the terminal ring in **Br_4TAP** and **I_4TAP** compared to **H_4TAP**.

#Ring	1	2	3	4	5
**Neutral**					
**H_4TAP(0)**	0.313	-0.418	0.212	-0.420	0.313
**Br_4TAP(0)**	0.259	-0.377	0.245	-0.390	0.263
**I_4TAP(0)**	0.261	-0.379	0.244	-0.391	0.265
**Anion**					
**H_4TAP(-1)**	0.124	-0.557	-0.122	-0.571	0.127
**Br_4TAP(-1)**	0.049	-0.517	-0.059	-0.523	0.051
**I_4TAP(-1)**	0.049	-0.517	-0.059	-0.523	0.051
**Dianion**					
**H_4TAP(-2)**	-0.042	-0.711	-0.492	-0.713	-0.042
**Br_4TAP(-2)**	-0.137	-0.656	-0.412	-0.660	-0.136
**I_4TAP(-2)**	-0.119	-0.678	-0.406	-0.678	-0.120

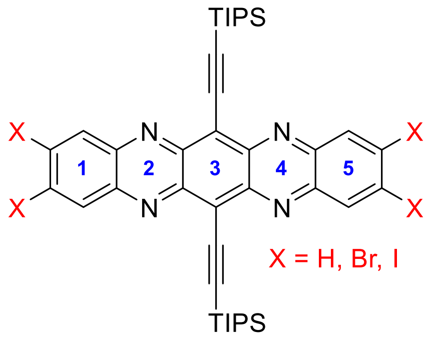

6.4 Absorption Spectra

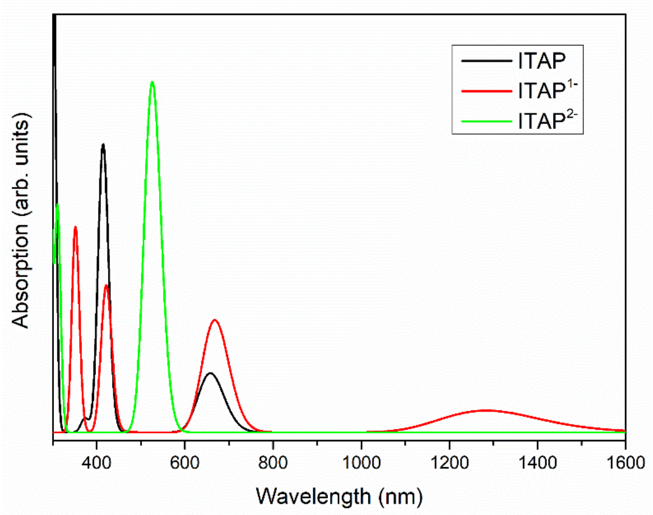

Figure S 24. Calculated UV-vis-NIR absorption spectra of **I_4TAP** in its neutral (black), monoanionic (red), and dianionic forms (green).

6.5 Nature of Iodine-Iodine Interactions in Crystals

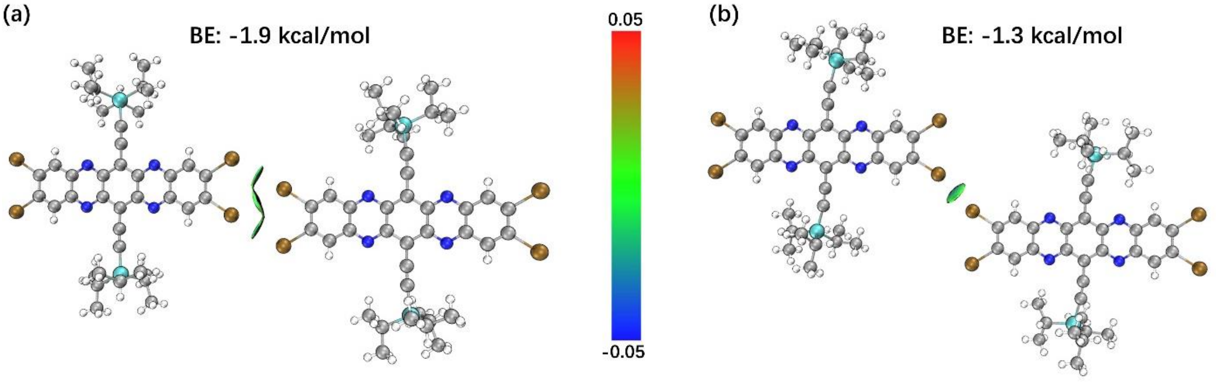

Figure S 25. Intermolecular interaction analyses for DM-A and DM-B, and the corresponding isosurface by IGMH (isovalue = 0.005 a.u.), where blue, green, and red represent strong interaction, weak interaction, and steric effect.

As shown in Figure S 25, the BSSE corrected BE of DM-A was computed to be -1.9 kcal/mol, which is stronger than that of DM-B (-1.3 kcal/mol). The isosurface of IGMH analysis revealed that the major intermolecular interaction regions correspond to the weak iodine-iodine interactions (green area). It can be also seen that there is a more extensive green isosurface in DM-A than that in DM-B, due to the multiple iodine-iodine interactions.

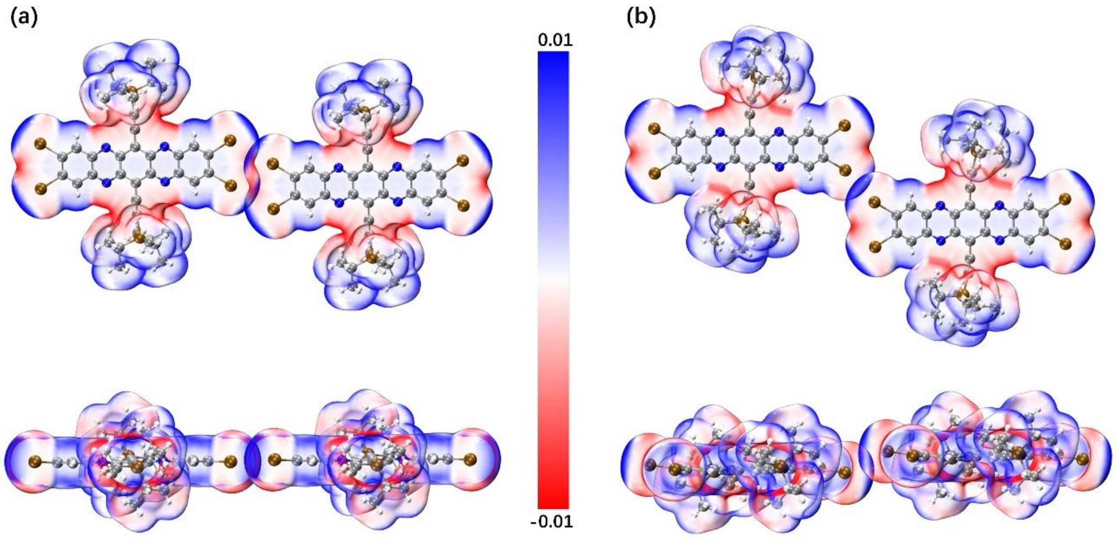

Figure S 26. Electrostatic potential (ESP) mapped on van der Waals (vdW) surface of each fragment in the systems of DM-A (a) and DM-B (b). Both top view and side view are shown. Red and blue represent electron-rich (negative value) and electron-deficient (positive value) regions, respectively.

Figure S 26 shows the ESP mapped on vdW surface of each fragment in the systems of DM-A and DM-B. One can see that all the iodine atoms have both positive (blue) and negative (red) ESP on their vdW surfaces. The positive region is essentially on the extension of the C−I bond, which overlaps with the negative region of the iodine atom in another fragment. Therefore, it is possible for the iodine atom on each side of the I_4TAP molecule to interact electrostatically with each other. This type of iodine-iodine interaction can be explained by the well-known σ-hole and σ-hole bonding concepts.[25]

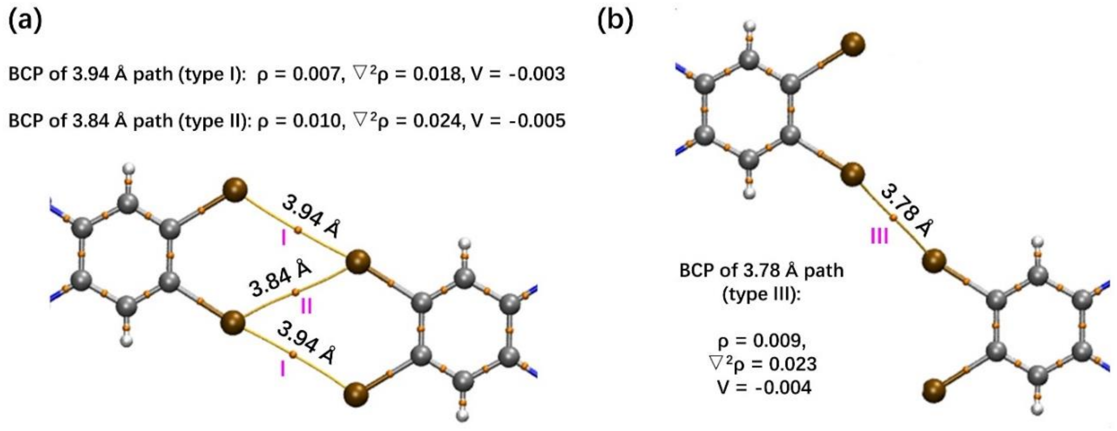

Figure S 27. AIM analyses for the interfragment interaction regions of DM-A (a) and DM-B (b). Orange points and yellow lines correspond to bond critical points (BCP) and interaction paths. The iodine-iodine distances and the parameters of the BCP (in a.u) are also given, including ρ (total electron density), $\nabla^2\rho$ (Laplacian of the total electron density), and *V* (potential energy density).

In the framework of AIM analysis, the bond critical points (BCP, orange points) and the relative interaction paths (yellow lines) between the fragments in DM-A and DM-B are depicted in Figure S 27. Generally speaking, there are two types of iodine-iodine interactions in DM-A with iodine-iodine distances of 3.94 (type I) and 3.84 Å (type II), but only one type in DM-B with a distance of 3.78 Å (type III). The values of $\nabla^2\rho$ are positive for all the BCP, indicating a depletion of electron density. Thus, the iodine-iodine interactions could be characteristic of closed-shell interactions.[26] The electron density ρ(BCP) and potential energy density *V*(BCP) at the BCP can be used to describe the strength of interatomic interaction. Larger ρ(BCP) and more negative *V*(BCP) usually suggest stronger interaction. The order of ρ(BCP) is: I < III < II, which is not fully consistent with the order of the iodine-iodine distances. A similar conclusion can also be made for the trend of *V*(BCP).

Table S 5. Components of intermolecular interaction (in kcal/mol) between the monomers in DM-A and DM-B computed by scaled SAPT0/def2-TZVP.

	DM-A	DM-B
**Exchange**	11.1	5.0
**Electrostatics**	-4.5	-2.3
**Induction**	-1.8	-1.0
**Dispersion**	-8.7	-3.8
**Total interaction**	-3.9	-2.1

Table S5 lists the components of the total interaction energies for DM-A and DM-B computed by scaled SAPT0/def2-TZVP. For each dimer system, the sum of attractive interactions (electrostatic, induction, and dispersion) outcompetes the repulsive exchange contribution, resulting in the negative values of the total interaction energies. On the other hand, both the electrostatic and dispersion components make important contributions to the attractive interaction, while the induction shows a relatively small effect. It is also noted that the total interaction of DM-A obtained from scaled SAPT0/def2-TZVP calculations is more negative than that of DM-B (-3.9 *vs*. -2.1 kcal/mol), which is consistent with the trend of the BSSE corrected BE provided by ωB97X-D/def2-TZVP calculations (-1.9 *vs*. -1.3 kcal/mol).

Based on the above results and discussion, we conclude that the iodine-iodine interactions in crystals are of σ-hole interaction nature with considerable contributions from both electrostatic and dispersion components. Although the

iodine-iodine distance in structure A is slightly longer than that in structure B, the overall three iodine-iodine interactions of the former are stronger than the individual iodine-iodine interaction of the latter.

7. Photophysical Study

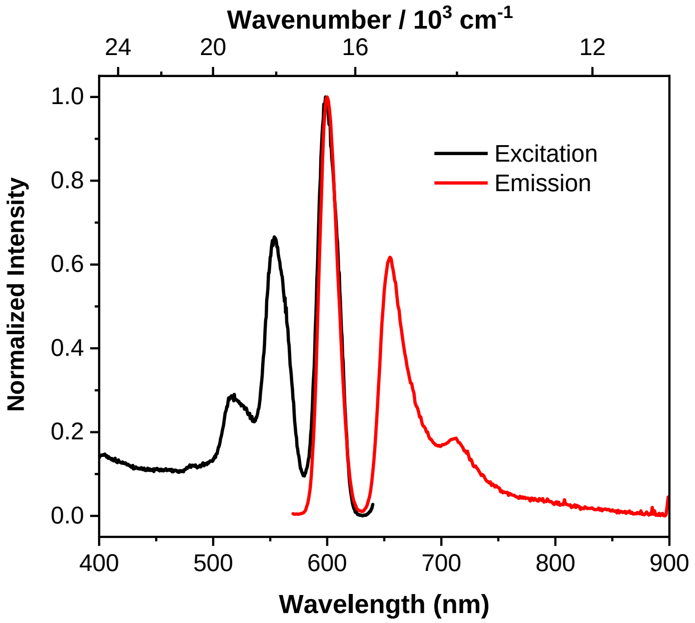

Figure S 28. Excitation and emission spectra of **I_4TAP^{2-}**.

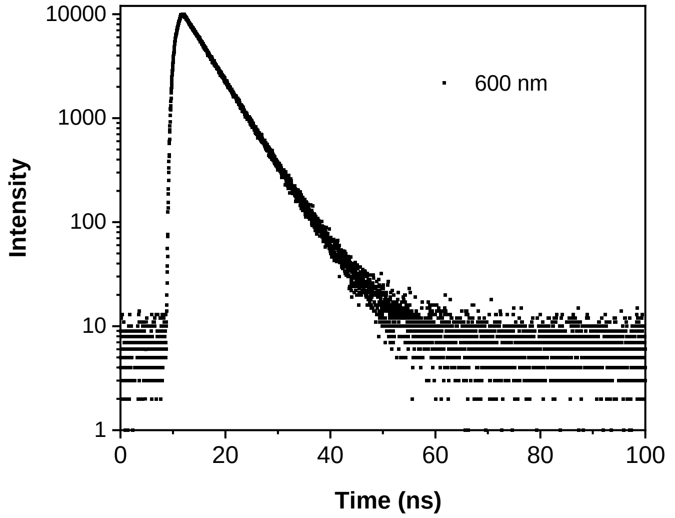

Figure S 29. Fluorescence decay (5.35 ns) of **I_4TAP^{2-}** dianion at 600 nm in dilute Et_2O solution.

8. Spectroelectrochemical Study

Spectra of the anions were recorded in reflection mode in a three-electrode custom-made cell (6 mm Pt-disc working electrode, Pt counter and pseudo reference electrode) implemented in an Agilent Cary 5000 UV-Vis-NIR spectrometer. The optical path was adjusted to 100 µm with a micrometer screw. Potentials were applied with a PAR 283 potentiostat (Princeton Applied Research). The substance concentration was ca. 2.5 x 10^{-5} M.

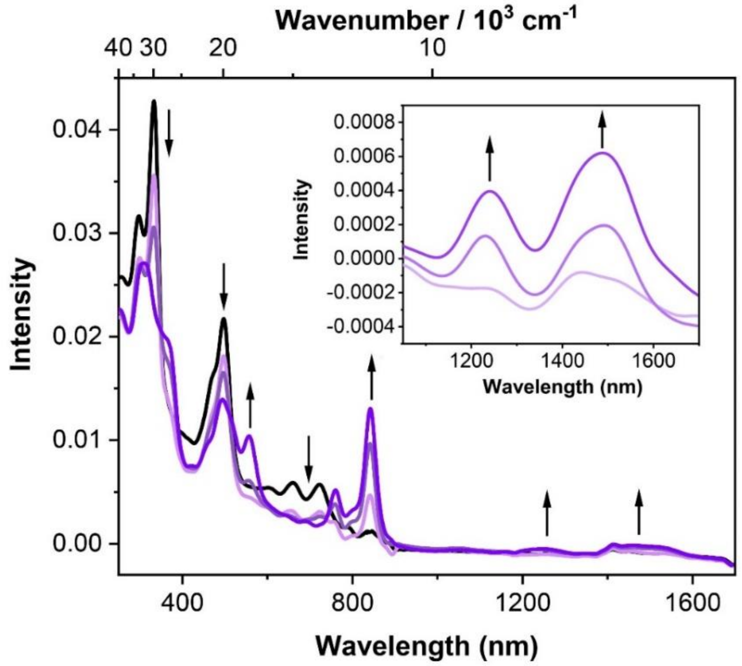

Figure S 30. **I$_4$TAP** is gradually reduced from the neutral compound to its monoanion. The inset displays changes in the long wavelength absorption region subtracted by the spectrum of the neutral compound to mitigate artifacts that arose due to the low concentration.

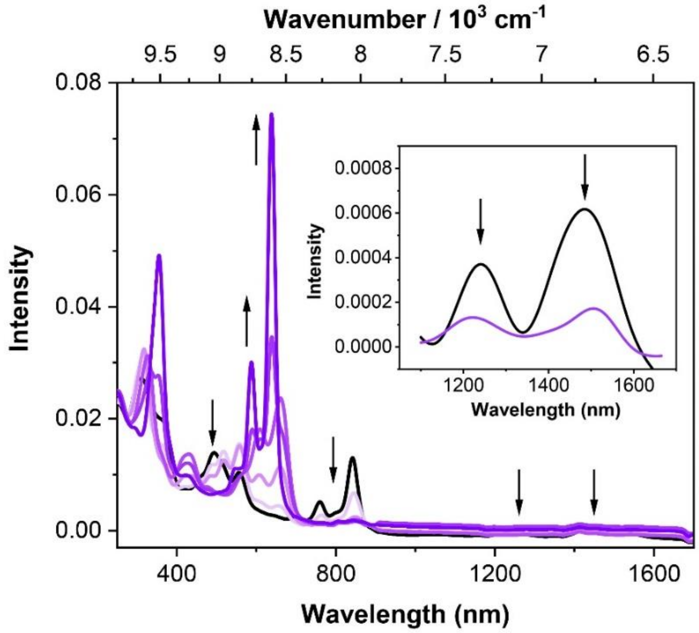

Figure S 31. **I$_4$TAP** is gradually reduced from monoanion to dianion. The inset displays changes in the long wavelength absorption region subtracted by the spectrum of the neutral compound to mitigate artifacts that arose due to the low concentration.

9. Stability Study

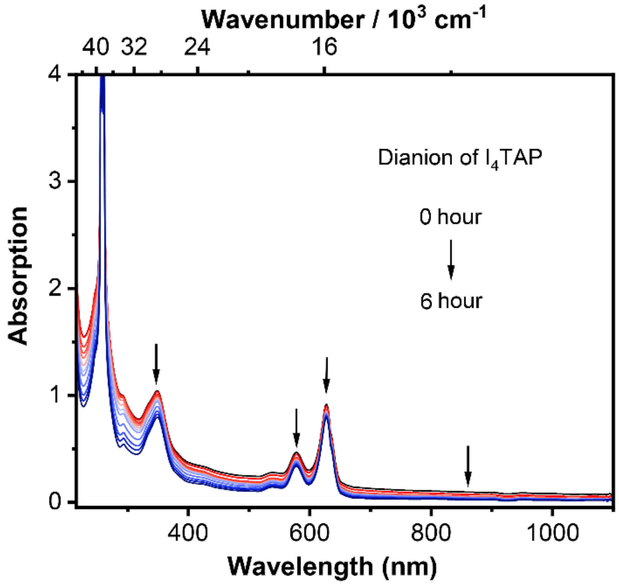

Figure S 32. UV-vis spectra of an Et_2O solution of **I_4TAP^{2-}** in air vs. time.

Solutions of the radical anion of **I_4TAP** are bright blue. When exposed to air, this color does not fade for a significant amount of time. Also, crystals of **$I_4TAP^{\bullet-}$** can be prepared for X-ray diffraction measurements (ca. 30 min) under ambient conditions, suggesting air stability.

10. Crystallographic Data

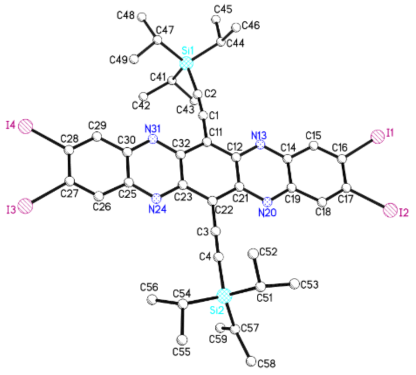

Table S 6. Crystal data and structure refinement for **I_4TAP** structure A (brickwall).

CCDC Entry	2155634	
Empirical formula	$C_{40}H_{46}I_4N_4Si_2 \cdot O_{0.20}$ (crystal water)	
Formula weight	1149.73	
Temperature	100(2) K	
Wavelength	1.54178 Å	
Crystal system	triclinic	
Space group	$P\bar{1}$	
Z	3	
Unit cell dimensions	*a* = 10.6183(18) Å	α = 110.634(13) deg.
	b = 18.084(3) Å	β = 94.523(14) deg.
	c = 18.919(3) Å	γ = 94.654(14) deg.
Volume	3366.0(10) $Å^3$	
Density (calculated)	1.71 g/cm^3	
Absorption coefficient	22.56 mm^{-1}	
Crystal shape	plank	
Crystal size	0.265 x 0.107 x 0.049 mm^3	
Crystal colour	black	
Theta range for data collection	2.5 to 68.2 deg.	
Index ranges	$-12 \leq h \leq 12$, $-21 \leq k \leq 18$, $-12 \leq l \leq 22$	
Reflections collected	36442	
Independent reflections	11896 (R(int) = 0.0298)	
Observed reflections	10785 ($I > 2\sigma(I)$)	
Absorption correction	Semi-empirical from equivalents	
Max. and min. transmission	2.78 and 0.22	
Refinement method	Full-matrix least-squares on F^2	
Data/restraints/parameters	11896 / 969 / 729	
Goodness-of-fit on F^2	1.08	
Final R indices ($I>2\sigma(I)$)	R1 = 0.042, wR2 = 0.118	
Largest diff. peak and hole	2.22 and -1.43 e$Å^{-3}$	

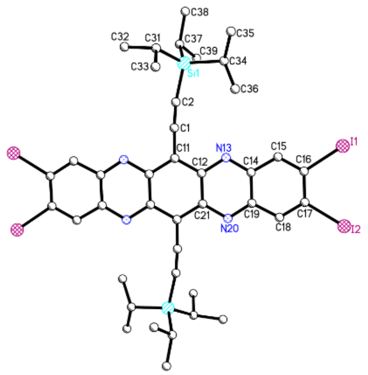

Table S 7. Crystal data and structure refinement for **I_4TAP** structure B (staircase) at 200 K.

CCDC entry	2155635	
Empirical formula	$C_{40}H_{46}I_4N_4Si_2$	
Formula weight	1146.59	
Temperature	200(2) K	
Wavelength	0.71073 Å	
Crystal system	triclinic	
Space group	$P\,\bar{1}$	
Z	1	
Unit cell dimensions	a = 7.6849(6) Å	α = 86.0743(11) deg.
	b = 9.8631(7) Å	β = 86.4298(12) deg.
	c = 14.5286(11) Å	γ = 86.3349(11) deg.
Volume	1094.54(14) $Å^3$	
Density (calculated)	1.74 g/cm^3	
Absorption coefficient	2.93 mm^{-1}	
Crystal shape	plank	
Crystal size	0.261 x 0.040 x 0.015 mm^3	
Crystal colour	green	
Theta range for data collection	1.4 to 30.2 deg.	
Index ranges	$-10 \le h \le 10$, $-13 \le k \le 13$, $-19 \le l \le 20$	
Reflections collected	22640	
Independent reflections	6005 (R(int) = 0.0286)	
Observed reflections	4831 ($I > 2\sigma(I)$)	
Absorption correction	Semi-empirical from equivalents	
Max. and min. transmission	0.97 and 0.86	
Refinement method	Full-matrix least-squares on F^2	
Data/restraints/parameters	6005 / 0 / 232	
Goodness-of-fit on F^2	1.02	
Final R indices ($I>2\sigma(I)$)	R1 = 0.026, wR2 = 0.050	
Largest diff. peak and hole	0.57 and -0.66 $eÅ^{-3}$	

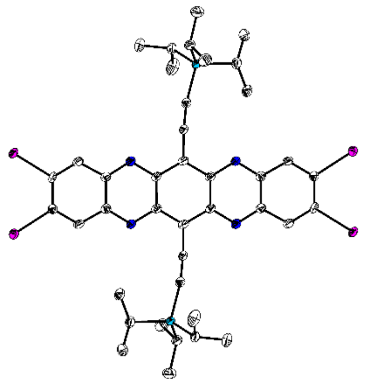

Table S 8. Crystal data and structure refinement for I_4**TAP** structure B (staircase) at 100 K.

CCDC entry	2133503	
Empirical formula	$C_{40}H_{46}I_4N_4Si_2$	
Formula weight	1146.59	
Temperature	100(2) K	
Wavelength	1.54184 Å	
Crystal system	triclinic	
Space group	$P\,\overline{1}$	
Z	1	
Unit cell dimensions	*a* =7.6086(3) Å	α = 85.967(3) deg.
	b =9.8005(3) Å	β = 86.606(3) deg.
	c =14.5291(6) Å	γ = 85.931(3) deg.
Volume	1076.39(7) $Å^3$	
Density (calculated)	1.77 g/cm^3	
Absorption coefficient	23.51 mm^{-1}	
Crystal shape	needle	
Crystal size	0.353 x 0.067 x 0.037 mm^3	
Crystal colour	green	
Theta range for data collection	3.0 to 74.5 deg.	
Index ranges	$-9\leq h\leq 8$, $-12\leq k\leq 12$, $-18\leq l\leq 18$	
Reflections collected	21714	
Independent reflections	4385 (R(int) = 0.0680)	
Observed reflections	3985 ($I > 2\sigma(I)$)	
Absorption correction	Semi-empirical from equivalents	
Max. and min. transmission	0.77 and 0.04	
Refinement method	Full-matrix least-squares on F^2	
Data/restraints/parameters	4385 / 0 / 232	
Goodness-of-fit on F^2	1.05	
Final R indices ($I>2\sigma(I)$)	R1 = 0.044, wR2 = 0.121	
Largest diff. peak and hole	2.00 and -1.90 $eÅ^{-3}$	

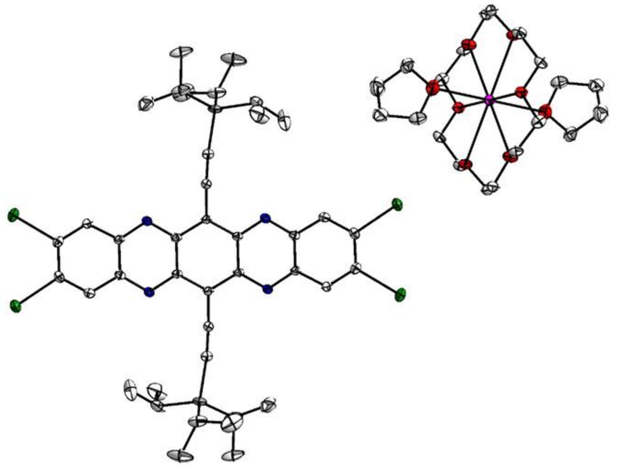

Table S 9. Crystal data and structure refinement for **I_4TAP^-.**

CCDC entry	2133504	
Empirical formula	$C_{40}H_{46}I_4N_4Si_2 \cdot C_{20}H_{40}KO_8 \cdot 2(C_4H_8O)$	
Formula weight	1738.41	
Temperature	100(2) K	
Wavelength	0.71073 Å	
Crystal system	monoclinic	
Space group	$P2_1/n$	
Z	2	
Unit cell dimensions	a = 11.445(9) Å	α = 90 deg.
	b = 17.953(10) Å	β = 97.68(2) deg.
	c = 18.713(12) Å	γ = 90 deg.
Volume	3810(4) Å^3	
Density (calculated)	1.515 g/cm^3	
Absorption coefficient	1.776 mm^{-1}	
Crystal shape	block	
Crystal size	0.454 × 0.334 × 0.316 mm^3	
Crystal colour	red	
Theta range for data collection	2.4 to 29.7 deg.	
Index ranges	$-15\leq h\leq15, -24\leq k\leq23, -26\leq l\leq23$	
Reflections collected	45275	
Independent reflections	10709 (R(int) = 0.0574)	
Observed reflections	9376 ($I > 2\sigma(I)$)	
Absorption correction	Semi-empirical from equivalents	
Max. and min. transmission	0.75 and 0.32	
Refinement method	Full-matrix least-squares on F^2	
Data/restraints/parameters	10709 / 270 / 455	
Goodness-of-fit on F^2	1.05	
Final R indices ($I>2\sigma(I)$)	R1 = 0.036, wR2 = 0.092	
Largest diff. peak and hole	1.76 and -1.02 eÅ^{-3}	

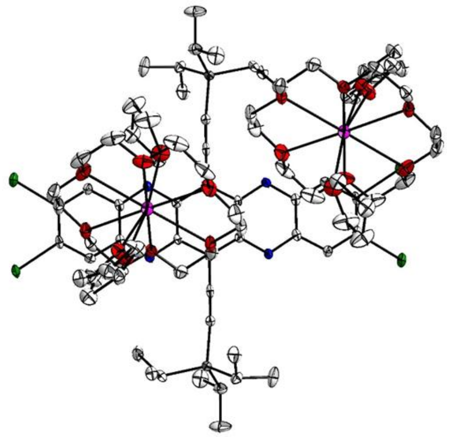

Table S 10. Crystal data and structure refinement for **I_4TAP^{2-}** polymorph A.

CCDC entry	2133505	
Empirical formula	$C_{40}H_{46}I_4N_4Si_2 \cdot 2(C_{20}H_{40}KO_8) \cdot C_5H_{12}$	
Formula weight	2113.97	
Temperature	100(2) K	
Wavelength	0.71073 Å	
Crystal system	triclinic	
Space group	$P\,\overline{1}$	
Z	1	
Unit cell dimensions	a = 12.5861(13) Å	α = 100.426(2) deg.
	b = 14.3108(15) Å	β = 113.678(2) deg.
	c = 15.1946(15) Å	γ = 91.053(2) deg.
Volume	2452.4(4) Å^3	
Density (calculated)	1.431 g/cm^3	
Absorption coefficient	1.439 mm^{-1}	
Crystal shape	block	
Crystal size	0.267 × 0.206 × 0.187 mm^3	
Crystal colour	red	
Theta range for data collection	2.1 to 26.5 deg.	
Index ranges	$-15 \le h \le 15$, $-17 \le k \le 17$, $-19 \le l \le 19$	
Reflections collected	35057	
Independent reflections	10094 (R(int) = 0.0425)	
Observed reflections	7766 ($I > 2\sigma(I)$)	
Absorption correction	Semi-empirical from equivalents	
Max. and min. transmission	0.75 and 0.63	
Refinement method	Full-matrix least-squares on F^2	
Data/restraints/parameters	10094 / 354 / 586	
Goodness-of-fit on F^2	1.01	
Final R indices ($I>2\sigma(I)$)	R1 = 0.036, wR2 = 0.087	
Largest diff. peak and hole	1.21 and -0.95 eÅ^{-3}	

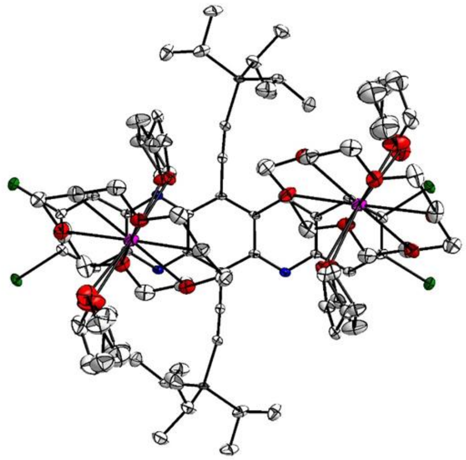

Table S 11. Crystal data and structure refinement for **I_4TAP^{2-}** polymorph B.

CCDC entry	2133507	
Empirical formula	$C_{40}H_{46}I_4N_4Si_2 \cdot 2(C_{20}H_{40}KO_8) \cdot 4(C_4H_8O)$	
Formula weight	2330.23	
Temperature	100(2) K	
Wavelength	0.71073 Å	
Crystal system	triclinic	
Space group	$P\overline{1}$	
Z	1	
Unit cell dimensions	a = 12.926(7) Å	α = 63.903(12) deg.
	b = 15.995(7) Å	β = 69.29(2) deg.
	c = 16.425(7) Å	γ = 67.28(2) deg.
Volume	2743(2) Å^3	
Density (calculated)	1.411 g/cm^3	
Absorption coefficient	1.297 mm^{-1}	
Crystal shape	block	
Crystal size	0.305 × 0.28 × 0.166 mm^3	
Crystal colour	red	
Theta range for data collection	1.1 to 28.0 deg.	
Index ranges	$-17 \leq h \leq 10$, $-21 \leq k \leq 20$, $-21 \leq l \leq 21$	
Reflections collected	71689	
Independent reflections	13210 (R(int) = 0.0555)	
Observed reflections	9986 ($I > 2\sigma(I)$)	
Absorption correction	Semi-empirical from equivalents	
Max. and min. transmission	0.75 and 0.64	
Refinement method	Full-matrix least-squares on F^2	
Data/restraints/parameters	13210 / 480 / 675	
Goodness-of-fit on F^2	1.06	
Final R indices ($I>2\sigma(I)$)	R1 = 0.045, wR2 = 0.107	
Largest diff. peak and hole	1.99 and -1.09 eÅ^{-3}	

11. References

[1] G. R. Fulmer, A. J. Miller, N. H. Sherden, H. E. Gottlieb, A. Nudelman, B. M. Stoltz, J. E. Bercaw, K. I. Goldberg, *Organometallics* **2010**, *29*, 2176-2179.
[2] G. M. Sheldrick, *Acta Crystallogr., Sect. A: Found. Adv*. **2015**, *A71*, 3-8.
[3] G. M. Sheldrick, *Acta Crystallogr., Sect. C: Struct. Chem*. **2015**, *C71*, 3-8.
[4] C. B. Hübschle, G. M. Sheldrick, and B. Dittrich, *J. Appl. Cryst.* **2011**, *44*, 1281-1284.
[5] H. Putz and K. Brandenburg GbR, Diamond Version 4.2.0. Crystal and Molecular Structure Visualization, Crystal Impact, Kreuzherrenstr. 102, 53227 Bonn, Germany.
[6] J. D. Kinder, W. J. Youngs, *Organometallics* **1996**, *15*, 460-463.
[7] L. Ji, A. Friedrich, I. Krummenacher, A. Eichhorn, H. Braunschweig, M. Moos, S. Hahn, F. L. Geyer, O. Tverskoy, J. Han, C. Lambert, A. Dreuw, T. B. Marder, U. H. F. Bunz, *J. Am. Chem. Soc.* **2017**, *139*, 15968-15976.
[8] D. Liu, Z. He, Y. Su, Y. Diao, S.C.B. Mannsfeld, Z. Bao, J. Xu, Q. Miao. *Adv. Mater.* **2014**, *26*: 7190-7196.
[9] a) M. Chu, J. X. Fan, S. Yang, D. Liu, C. F. Ng, H. Dong, A. M. Ren, Q. Miao, *Adv. Mater.* **2018**, *30*, e1803467. b) X. Xu, Y. Yao, B. Shan, X. Gu, D. Liu, J. Liu, J. Xu, N. Zhao, W. Hu, Q. Miao, *Adv. Mater.* **2016**, *28*, 5276-5283.
[10] H. H. Choi, K. Cho, C. D. Frisbie, H. Sirringhaus, V. Podzorov, *Nat. Mater.* **2017**, *17*, 2-7.
[11] J. D. Chai, M. Head-Gordon, *Phys. Chem. Chem. Phys.* **2008**, *10*, 6615-6620.
[12] F. Weigend, R. Ahlrichs, *Phys. Chem. Chem. Phys.* **2005**, *7*, 3297-3305.
[13] D. Rappoport, F. Furche, *J. Chem. Phys.* **2010**, *133*, 134105.
[14] A. Dreuw, M. Head-Gordon, *Chem. Rev.* **2005**, *105*, 4009-4037.
[15] a) J. Tomasi, B. Mennucci, E. Cancès *J. Mol. Struct. THEOCHEM* **1999**, *464*, 211-226.
b) J. Tomasi, *Comp. Mol. Sci.* **2011**, *1*, 855-867.
[16] Cammi, R., Bonaccorsi, R., Tomasi, J. *Theor. Chim. Acta* **1985**, *68*, 271–283.
[17] T. Lu, Q. Chen, *J. Comput. Chem.* **2022**, *43*, 539–555.
[18] J. Zhang, T. Lu, *Phys. Chem. Chem. Phys.* **2021**, *23*, 20323–20328.
[19] F. W. Bader, Atoms in Molecules: A Quantum Theory, *Oxford University Press: New York*, **1994**.
[20] T. Lu, F.J. Chen, *Comput. Chem.* **2012**, *33*, 580–592.
[21] Humphrey, W., Dalke, A. & Schulten, K. *J. Mol. Graph.* **1996,** *14*, 33–38.
[22] a) E. G. Hohenstein, C. D. Sherrill, *Comp. Mol. Sci.* **2012**, *2*, 304–326. b) T.M. Parker, L.A. Burns, R.M. Parrish, A.G. Ryno, C.D. Sherrill, *J. Chem. Phys.* **2014**, *140*, 094106.
[23] D. G. A. Smith, L. A. Burns, A. C. Simmonett, R. M. Parrish, M. C. Schieber, R. Galvelis, P. Kraus, H. Kruse, R. Di Remigio, A. Alenaizan, A. M. James, S. Lehtola, J. P. Misiewicz, M. Scheurer, R. A. Shaw, J. B. Schriber, Y. Xie, Z. L. Glick, D. A. Sirianni, J. S. O'Brien, J. M. Waldrop, A. Kumar, E. G. Hohenstein, B. P. Pritchard, B. R. Brooks, H. F. Schaefer, 3rd, A. Y. Sokolov, K. Patkowski, A. E. DePrince, 3rd, U. Bozkaya, R. A. King, F. A. Evangelista, J. M. Turney, T. D. Crawford, C. D. Sherrill, *J. Chem. Phys.* **2020**, *152*, 184108.
[24] Gaussian 16, Revision C.01, M. J. Frisch, G. W. Trucks, H. B. Schlegel, G. E. Scuseria, M. A. Robb, J. R. Cheeseman, G. Scalmani, V. Barone, G. A. Petersson, H. Nakatsuji, X. Li, M. Caricato, A. V. Marenich, J. Bloino, B. G. Janesko, R. Gomperts, B. Mennucci, H. P. Hratchian, J. V. Ortiz, A. F. Izmaylov, J. L. Sonnenberg, D. Williams-Young, F. Ding, F. Lipparini, F. Egidi, J. Goings, B. Peng, A. Petrone, T. Henderson, D. Ranasinghe, V. G. Zakrzewski, J. Gao, N. Rega, G. Zheng, W. Liang, M. Hada, M. Ehara, K. Toyota, R. Fukuda, J. Hasegawa, M. Ishida, T. Nakajima, Y. Honda, O. Kitao, H. Nakai, T. Vreven, K. Throssell, J. A. Montgomery, Jr., J. E. Peralta, F. Ogliaro, M. J. Bearpark, J. J. Heyd, E. N. Brothers, K. N. Kudin, V. N. Staroverov, T. A. Keith, R. Kobayashi, J. Normand, K. Raghavachari, A. P. Rendell, J. C. Burant, S. S. Iyengar, J. Tomasi, M. Cossi, J. M. Millam, M. Klene, C. Adamo, R. Cammi, J. W. Ochterski, R. L. Martin, K. Morokuma, O. Farkas, J. B. Foresman, and D. J. Fox, Gaussian, Inc., Wallingford CT, **2016**.
[25] P. Politzer, J.S. Murray, M.C. Concha, *J. Mol. Model.* **2008**, *14*, 659–665.
[26] D. J. R. Duarte, G. L. Sosa, N. M. Peruchena, *J. Mol. Model.* **2013**, *19*, 2035–2041.
